# MUC1-C Dictates PBRM1-Mediated Chronic Induction of Interferon Signaling, DNA Damage Resistance, and Immunosuppression in Triple-Negative Breast Cancer

**DOI:** 10.1158/1541-7786.MCR-22-0772

**Published:** 2022-11-29

**Authors:** Nami Yamashita, Yoshihiro Morimoto, Atsushi Fushimi, Rehan Ahmad, Atrayee Bhattacharya, Tatsuaki Daimon, Naoki Haratake, Yuka Inoue, Satoshi Ishikawa, Masaaki Yamamoto, Tsuyoshi Hata, Sayuri Akiyoshi, Qiang Hu, Tao Liu, Henry Withers, Song Liu, Geoffrey I. Shapiro, Tomoharu Yoshizumi, Mark D. Long, Donald Kufe

**Affiliations:** 1 Department of Medical Oncology, Dana-Farber Cancer Institute Harvard Medical School, Boston, Massachusetts.; 2 Department of Surgery and Science, Graduate School of Medical Sciences, Kyushu University, Fukuoka, Japan.; 3 Department of Biostatistics & Bioinformatics, Roswell Park Comprehensive Cancer Center, Buffalo, New York.

## Abstract

The polybromo-1 (PBRM1) chromatin-targeting subunit of the SWI/SNF PBAF chromatin remodeling complex drives DNA damage resistance and immune evasion in certain cancer cells through mechanisms that remain unclear. STAT1 and IRF1 are essential effectors of type I and II IFN pathways. Here, we report that MUC1-C is necessary for PBRM1 expression and that it forms a nuclear complex with PBRM1 in triple-negative breast cancer (TNBC) cells. Analysis of global transcriptional (RNA-seq) and chromatin accessibility (ATAC-seq) profiles further demonstrated that MUC1-C and PBRM1 drive STAT1 and IRF1 expression by increasing chromatin accessibility of promoter-like signatures (PLS) on their respective genes. We also found that MUC1-C, PBRM1, and IRF1 increase the expression and chromatin accessibility on PLSs of the (i) type II IFN pathway *IDO1* and *WARS* genes and (ii) type I IFN pathway *RIG-I*, *MDA5*, and *ISG15* genes that collectively contribute to DNA damage resistance and immune evasion. In support of these results, targeting MUC1-C in wild-type *BRCA* TNBC cells enhanced carboplatin-induced DNA damage and the loss of self-renewal capacity. In addition, MUC1-C was necessary for DNA damage resistance, self-renewal, and tumorigenicity in olaparib-resistant *BRCA1-*mutant TNBC cells. Analysis of TNBC tumors corroborated that (i) MUC1 and PBRM1 are associated with decreased responsiveness to chemotherapy and (ii) MUC1-C expression is associated with the depletion of tumor-infiltrating lymphocytes (TIL). These findings demonstrate that MUC1-C activates PBRM1, and thereby chromatin remodeling of IFN-stimulated genes that promote chronic inflammation, DNA damage resistance, and immune evasion.

**Implications:**

MUC1-C is necessary for PBRM1-driven chromatin remodeling in chronic activation of IFN pathway genes that promote DNA damage resistance and immunosuppression.

## Introduction

The type I IFN pathway is chronically activated in cancer cells by DNA damage-associated molecular patterns that are generated in part by genomic instability (1). The cyclic GMP-AMP (cGAMP) synthase (cGAS)-stimulator of IFN genes (STING) recognizes accumulation of DNA in the cytosol (2, 3). Other pattern-recognition receptors (PRR) that recognize cytosolic RNA, including RIG-I and MDA5 (1), can function as non-redundant RNA sensors. Stimulation of these PRRs induces the production of type I IFNs (IFNα and IFNβ) and activation of genes with IFN-stimulated response elements (ISRE; ref. 1). Oncogene-driven replicative stress and activation of the IFN-related DNA damage resistance signature (IRDS) are linked to chronic production of low levels of type I IFNs (4, 5). The type II IFN pathway is stimulated by IFNγ and drives the formation of STAT1 homodimers that bind directly to DNA at gamma-activated sequences and activate IFN-stimulated genes (ISG) which play roles in immune surveillance and immune evasion (6, 7). IRF1 regulates the expression of ISGs by binding directly to the ISRE or IRF response element (8). In cancer cells, STAT1 and IRF1 are important effectors of type I and II IFN stimulation and have the capacity to complement each other in the chronic activation of ISGs that promote DNA damage resistance and immune evasion (8, 9). The involvement of chromatin remodeling in the regulation of STAT1, IRF1, and type I and II IFN ISGs is not well understood.

The SWI/SNF PBAF chromatin remodeling complex includes the PBRM1, ARID2, and BRD7 subunits (10). PBRM1 contributes to transcriptional silencing for the repair of DNA double-strand breaks and maintaining genomic stability during mitosis (11). PBRM1 also plays a role in the regulation of genes involved in the DNA damage response and in maintaining redox balance (12–14). Inactivation of PBRM1 in human cancers thereby contributes to replication stress and confers synthetic lethality to DNA repair inhibitors targeting PARP and ATR (15). DNA damage is an important determinant of innate immune signaling and is activated by cytosolic DNA (16, 17). PBRM1 deficiency and the associated DNA damage sensitize certain cancer cells to immune checkpoint inhibitor (ICI) treatment (18–21). Other reports have shown that PBRM1 loss has a reduced or no significant association with responsiveness to ICIs (22, 23). These contradictory findings could be related to the effects of PBRM1 on IFN-regulated gene expression, which has been reported to be increased (18, 21), as well as decreased (22), in settings of PBRM1 loss. Surprisingly, little is known about the functional involvement of PBRM1 in integrating chromatin remodeling with DNA damage resistance and immune evasion in cancer cells.

The *MUC1* gene evolved in mammals to protect barrier tissues from the loss of homeostasis (24, 25). *MUC1* encodes (i) an N-terminal subunit (MUC1-N) that is shed from the apical cell surface into a protective mucous gel, and (ii) a transmembrane C-terminal (MUC1-C) subunit that activates inflammatory, remodeling, and repair pathways associated with wound repair (24, 25). Chronic MUC1-C activation by prolonged inflammatory cycles of epithelial cell damage and repair contributes to cancer progression (24). Along these lines, MUC1-C activates the inflammatory STAT3 and NF-κB transcription factors (TFs) in auto-inductive loops, which in turn increase MUC1-C expression (24). The MUC1-C→pSTAT3 pathway induces TWIST1 and the epithelial mesenchymal transition (EMT); ref. 26). MUC1-C→NF-kB signaling induces (i) ZEB1 and EMT, (ii) DNA methyltransferases (DNMTs) 1/3b, and (iii) the Polycomb Repressive Complexes (PRC) 1 and 2, linking EMT with methylation of DNA and histones (27). The MUC1-C→NF-kB pathway also induces PD-L1 and immune evasion 28). Epigenetic reprogramming is necessary for wound repair, stem cell memory and the cancer stem cell (CSC) state (29). In concert with promoting the CSC state (24), MUC1-C activates the SWI/SNF BAF chromatin remodeling complex (30) and thereby regulates chromatin accessibility at enhancers of stemness-associated genes (25, 31). Activation of MUC1-C and stemness in triple-negative breast cancer (TNBC) cells has been linked to immune evasion and DNA damage resistance (24, 32–34). The present study demonstrates that MUC1-C activates PBRM1 and that MUC1-C/PBRM1 complexes increase chromatin accessibility and expression of ISGs that promote chronic inflammation, DNA damage resistance, and immune evasion.

## Materials and Methods

### Cell culture

Human BT-549 *BRCA1* wild-type TNBC (CVCL_1092, ATCC) cells were cultured in RPMI-1640 medium (Thermo Fisher Scientific) containing 10% FBS (GEMINI Bio-Products), 100 μg/mL streptomycin, 100 U/mL penicillin, and 10 μg/mL insulin. Parental MDA-MB-436 *BRCA1* mutant TNBC (CVCL_0623, ATCC) and olaparib-resistant MDA-MB-436RR cells (35) were cultured in RPMI-1640 medium containing 10% FBS, 100 μg/mL streptomycin, and 100 U/mL penicillin. MDA-MB-436RR cells were maintained in the presence of 5 μmol/L olaparib (Selleck Chemicals), which was removed 2 weeks before use (35). Cells were also treated with carboplatin (CBDCA; MilliporeSigma), olaparib, and GO-203 (24). Cell authentication was performed using short tandem repeat analysis every 3–4 months. The cells were monitored for mycoplasma contamination every 3–4 months using the MycoAlert *Mycoplasma* Detection Kit (Lonza). Cells were maintained in culture for 3–4 months for performing experiments.

### Mammosphere formation assay

Cells (2.5–5×10^3^) were seeded per well in 6-well ultra-low attachment culture plates (Corning) using the MammoCult Human Medium Kit (Stemcell Technologies). The mammospheres were (i) treated with vehicle or 500-ng/mL DOX (doxycycline), (ii) left untreated or treated with GO-203, (iii) treated with vehicle or carboplatin, and (iv) treated with vehicle or olaparib. Mammospheres with diameters >100 μm were counted in triplicate under an inverted microscope.

### Gene silencing and rescue

MUC1shRNA (MISSION shRNA TRCN0000122938) and a control scrambled shRNA (CshRNA; Millipore Sigma) were inserted into pLKO-tet-puro (Addgene_21915; Addgene) as described (36). CshRNA, MUC1shRNA, MUC1shRNA#2 (MISSION shRNA TRCN0000430218), IRF1shRNA (MISSION shRNA TRCN0000014672), IRF1shRNA#2 (MISSION shRNA TRCN0000218951), PBRM1shRNA (MISSION shRNA TRCN0000235890), and PBRM1shRNA#2 (MISSION shRNA TRCN0000235889) were produced in HEK293T (CVCL_0063, ATCC) cells as described (37). Flag-tagged MUC1-CD (38) was inserted into pInducer20 (Addgene_44012, Addgene). Cells transduced with the vectors were selected for growth in 1–2 μg/mL puromycin. For inducible gene silencing, the cells were treated with 0.1% DMSO as the vehicle control or 500-ng/mL DOX (Millipore Sigma).

### qRT-PCR

Total RNA was isolated using TRizol (Invitrogen). cDNAs was synthesized using the High Capacity cDNA Reverse Transcription Kit (Applied Biosystems) as previously described (36). Samples were amplified using Power SYBR Green PCR Master Mix (Applied Biosystems) and a CFX96 Touch Real-Time PCR Detection System (SCR_018064, Bio-Rad Laboratories). The primers used for qRT-PCR analysis are listed in Supplementary Table S1.

### Immunoblot analysis

Whole-cell lysates were prepared in RIPA buffer containing protease inhibitor cocktail (Thermo Fisher Scientific) as described (36). Immunoblotting was performed with anti–MUC1-C (#MA5–11202, 1:100; Thermo Fisher Scientific), anti-PBRM1 (A301–591A, 1:10,000; Bethyl Laboratories), anti-IRF1 [#8478, 1:1,000; Cell Signaling Technology (CST)], anti-STAT1 (#9172, 1:1,000; CST), anti-IDO1 (#86630S, 1:1,000 dilution; CST), anti-WARS (GTX110223, 40037, 1:1,000; GeneTex), anti–RIG-I (#3743, 1:1,000; CST), anti-MDA5 (#5321, 1:1,000; CST), anti-ISG15 (sc-166755, 1:250; Santa Cruz, Santa Cruz Biotechnology), anti-γH2AX (#9718, 1:1,000 dilution; CST), anti–β-actin (A5441; 1:50,000; Sigma), and anti-GAPDH (#2118, 1:1,000; CST).

### Co-immunoprecipitation of the nuclear proteins

Nuclear lysates were isolated as described (39). Nuclear proteins were incubated with anti–MUC1-C (#MA5–11202; Thermo Fisher Scientific), precipitated with Dynabeads Protein G (10003D; Thermo Fisher Scientific) and analyzed as described (36).

### Direct protein–binding assays

GST, GST-IRF1 (full-length; aa 1–325), GST-IRF1-N(aa 1–163), GST-IRF1-C(aa 163–325), GST-MUC1-CD (full-length; aa 1–72), GST-MUC1-CD(aa 1–45), GST-MUC1-CD(aa 46–72), and GST-MUC1-CD(AQA) were prepared as described (40). Purified GST-MUC1-CD was cleaved with thrombin to remove GST. Binding assays with GST fusion proteins and MUC1-CD or GST-IRF1 were performed for 2 hours at room temperature. Adsorbates on glutathione-conjugated beads were detected by immunoblotting.

### Chromatin immunoprecipitation

Chromatin immunoprecipitation (ChIP) was performed on cells crosslinked with 1% formaldehyde for 5 minutes at 37°C, quenched with 2 mol/L glycine, washed with PBS, and sonicated in a Covaris E220 sonicator to generate 300–600 bp DNA fragments, as described (41). Immunoprecipitation was performed using control IgG (Santa Cruz Biotechnology), anti–MUC1-C (#MA5–11202, Thermo Fisher Scientific), anti-PBRM1 (A301–591A; Bethyl Laboratories), and anti-IRF1 (#8478; CST). Precipitated DNAs were detected by PCR using the primers listed in Supplementary Table S2. The immunoprecipitated DNA was quantified using SYBR-green and the CFX96 Touch Real-Time PCR Detection System (Bio-Rad). Data are reported as fold enrichment relative to IgG levels.

### Chromatin accessibility assay

DNase I chromatin accessibility assays were performed as described (31). The DNA was purified and amplified by qPCR using the primers listed in Supplementary Table S2.

### Drug sensitivity and cell proliferation assays

MDA-MB-436 and MDA-MB-436RR cells were seeded at a density of 6,000 cells per well in 96-well plates. After 24 hours, cells were treated with different concentrations of olaparib. Cell viability was assessed using the Alamar Blue assay (Thermo Fisher Scientific) in sextuplicate wells. The IC_50_ value was determined by nonlinear regression of the dose–response data using Prism 9.0 (SCR_002798, GraphPad Software). Cell proliferation was assessed using the Alamar Blue assay (Thermo Fisher Scientific). Fluorescence intensity (560 nm excitation/590 nm emission) was measured in sextuplicate.

### RNA-seq analysis

Total RNA from cells cultured in triplicate was used to generate RNA-seq datasets, as described (31). Raw sequencing reads were aligned as described (42). Raw feature counts were normalized and analyzed using DESeq2 (SCR_015687) as described (43). Differential expression rank order was performed using Gene Set Enrichment Analysis (GSEA) as described (44). Gene set variation analysis (GSVA) was performed using the GSVA package (45). Gene sets queried included those from the Hallmark, Reactome, and Gene Ontology Biological Processes (GO-BP) Gene Sets available through the Molecular Signatures Database (MSigDB; ref. 46). A set of IFN response genes were identified to examine IFN signaling in *in vitro* and publicly available TNBC cohort data using the common genes found in the HALLMARK IFN_Gamma_Response and IFN_Alpha_Response pathways.

### ATAC-seq

ATAC-seq libraries were generated from three biologically independent replicates per condition as described (31). The accessibility of chromatin was explored using Integrative Genomics Viewer (IGV_2.13.0).

### Immunofluorescence analysis of γH2AX expression in mammospheres

Mammospheres were fixed with 4% paraformaldehyde (Sigma-Aldrich) at room temperature for 15 minutes. Samples were incubated with 1% Triton X-100 (Sigma-Aldrich) at room temperature for 10 minutes and blocked with 5% normal goat serum (Gibco). The mammospheres were attached to slides via cytospin at low speed (Shandon Cytospin 3; Shandon Scientific) and stained with anti-γH2AX (#9718, 1:400 dilution, CST) and goat anti-rabbit IgG H and L labeled with Alexa Fluor 488 (Abcam) as described (41). Nuclei were stained with ProLong Gold Antifade Mountant with DAPI (Invitrogen). Cells were imaged using a Leica THUNDER Imager 3D Cell Culture microscope, as described (41).

### Mouse tumor model studies

Six-week-old female nude mice (The Jackson Laboratory) were injected subcutaneously into the flank with 5×10^6^ tumor cells in 100 μL of a 1:1 solution of medium and Matrigel (BD Biosciences). When the mean tumor volume reached 100–150 mm^3^, the mice were pair-matched into groups and treated intraperitoneally with PBS or GO-203 (12 μg/g body weight) daily. Tumor measurements and body weights were recorded twice per week. These studies were conducted in accordance with the ethical regulations required for approval by the Dana-Farber Cancer Institute Animal Care and Use Committee (IACUC) under protocol 03–029.

### IHC analysis of TNBC samples

All patients consented to an institutional review board-approved research protocol in Department of Surgery and Science, Kyushu University (Japan), allowing specimen collection and clinical data. Written informed consent was obtained from each patient, and the study was conducted using anonymized data in accordance with recognized ethical guidelines. Core needle biopsy specimens were obtained from 21 patients with TNBC. Formalin-fixed, paraffin-embedded sections were deparaffinized in xylene and graded concentrations of ethanol and distilled water. Antigen retrieval was performed in citrate buffer (pH 6.0; C9999, Sigma-Aldrich). Slides were incubated with anti–MUC1-C (#16564, 1:200, CST) for 8 hours at 4°C, and a MACH 4 Universal HRP-Polymer Detection System (Biocare Medical) was used for detection. Immunostained sections were counterstained with hematoxylin. MUC1-C staining intensity (IS) and proportion (PS) in cancer cells were scored in a blinded manner and independently by two investigators (N. Yamashita and Y. Inoue). IS scored from 0 to 3. IS0, no staining; IS1, low staining; IS2, moderate staining; IS3, strong staining. PS scored from 0 to 4. PS0, no staining; PS1, <25%; S2, 25%–50%; PS3, 51%–75%, and PS4, >75%. The IHC score was calculated by multiplying the IS and PS (score range, 0–12). Stromal–tumor-infiltrating lymphocytes (sTIL) were evaluated according to guidelines from the International TILs Working Group (47). Stromal TILs occupying more than 50% of the total intratumoral stromal area were designated as high sTILs.

### Survival analysis of patients with TNBC

Survival curves based on MUC1 and PBRM1 expression levels were generated using the Kaplan–Meier Plotter (http://kmplot.com/analysis/; ref. 48), and the statistical difference was calculated using the log-rank test. Patients with breast cancer sorted by ER-negative, HER2-negative, and basal-phenotype (PAM50) and treated with chemotherapy were included in this analysis. A Cox proportional hazards regression model was used to assess the prognostic value of MUC1 and PBRM1 expression levels.

### Statistical analysis

Each experiment was performed at least three times. Unpaired two-tailed Student *t* tests were used to examine the differences between the mean ± SD of the two groups. *P* values were considered significant at *P* < 0.05. GraphPad Prism9 was used for all statistical analyses. Asterisks represent *, *P* ≤ 0.05; **, *P* ≤ 0.01; ***, *P* ≤ 0.001; ****, *P* ≤ 0.0001 with CI = 95%.

### Analysis of publicly available TNBC cohort datasets

TCGA-BRCA and METABRIC expression and clinical annotations of TNBC cohorts were obtained from the Genomic Data Commons data portal, processed via TCGAbiolinks package in R using TCGAWorkflow-guided practices, and analyzed as described previously (36).

### Data availability

All RNA-seq data reported here are available from the NCBI Gene Expression Omnibus (GEO, SCR_005012). BT-549 cells ± MUC1shRNA were previously deposited under GEO accession GSE206212. BT-549 cells ± IRF1shRNA (GSE212168), ±PBRM1shRNA (GSE212169), and MDA-MB-436 cells ± MUC1shRNA (GSE212587) were deposited in GEO SuperSeries accession GSE212170.

## Results

### MUC1-C regulates chromatin accessibility and activation of *IRF1* and *STAT1* in an auto-inductive circuit

**Figure 1.**
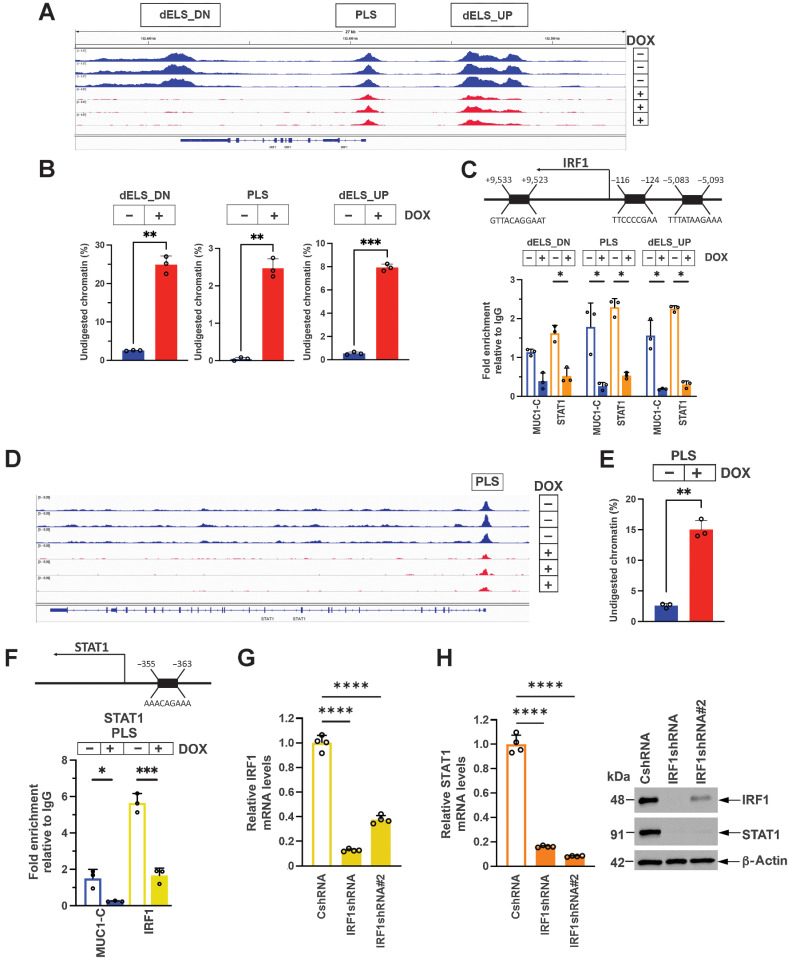
MUC1-C drives chromatin accessibility and activation of *IRF1* and *STAT1*. **A,** Genome browser snapshots of ATAC-seq data from the *IRF1* gene in BT-549/tet-MUC1shRNA cells treated with vehicle or DOX for 7 days. **B,** Chromatin was analyzed for accessibility by nuclease digestion. The results are expressed as the percentage of undigested chromatin (mean±SD and individual values). **C,** Schema of the *IRF1* gene highlighting positioning of a promoter-like signature (PLS) and distal enhancer-like signatures (dELS) downstream (dELS-DN) and upstream (dELS-UP) to the TSS. Soluble chromatin from BT-549/tet-MUC1shRNA cells treated with vehicle or DOX for 7 days was precipitated with a control IgG, anti–MUC1-C and anti-STAT1. The DNA samples were amplified by qPCR with primers for the indicated *IRF1* regions. The results (mean ± SD and individual values) are expressed as fold-enrichment as compared with that obtained from control IgG-precipitated chromatin (assigned a value of 1). **D** and **E,** Genome browser snapshot of ATAC-seq data from the *STAT1* PLS region in BT-549/tet-MUC1shRNA cells treated with vehicle or DOX for 7 days (**D**). Chromatin was analyzed for accessibility by nuclease digestion (**E**). The results are expressed as the percentage of undigested chromatin (mean ± SD and individual values). **F,** Schema of the *STAT1* gene with localization of a PLS upstream to the TSS. Soluble chromatin from BT-549/tet-MUC1shRNA cells treated with vehicle or DOX for 7 days was precipitated with a control IgG, anti–MUC1-C and anti-IRF1. The DNA samples were amplified by qPCR with primers for the *STAT1* PLS region. The results (mean±SD and individual values) are expressed as relative fold enrichment as compared with that obtained with IgG (assigned a value of 1). **G,** BT-549/CshRNA, BT-549/IRF1shRNA and BT-549/IRF1shRNA#2 cells were analyzed for IRF1 and STAT1 mRNA levels by qRT-PCR. The results (mean ± SD and individual values) are expressed as relative mRNA levels as compared with that obtained in CshRNA cells (assigned a value of 1). **H,** Lysates were immunoblotted with antibodies against the indicated proteins. *, *P* ≤ 0.05; **, *P* ≤ 0.01; ***, *P* ≤ 0.001; ****, *P* ≤ 0.0001.

STAT1 and IRF1 are important effectors of the type I and II IFN pathways (8, 9). MUC1-C binds directly to STAT1 and activates STAT1 target genes (5). *IRF1* contains an ISRE for STAT1 binding in a promoter-like signature (PLS; Fig. 1A; refs. 8, 49). As revealed by genome browser snapshots from ATAC-seq studies, MUC1-C silencing was associated with a decrease in chromatin accessibility at the *IRF1* PLS (Fig. 1A). We also found that MUC1-C silencing decreases chromatin accessibility at distal enhancer-like signatures (dELS) with putative ISREs upstream and downstream of the TSS (Fig. 1A). These changes in chromatin accessibility were confirmed by nuclease digestion assays (Fig. 1B), demonstrating that MUC1-C promotes chromatin opening at these sites. In support of these results, we found that MUC1-C and STAT1 occupy the PLS and dELS regions, and that silencing MUC1-C decreases their occupancy (Fig. 1C) and IRF1 expression (Supplementary Fig. S1A–S1D). Like *IRF1*, the *STAT1* PLS includes an ISRE, which was also found to be dependent on MUC1-C for opening chromatin (Fig. 1D and E). However, in contrast with the *IRF1* PLS, we found that the *STAT1* PLS is occupied by MUC1-C and IRF1 (Fig. 1F). Silencing MUC1-C decreased IRF1 occupancy of the *STAT1* PLS (Fig. 1F) and STAT1 expression, as confirmed with a different MUC1shRNA#2 and rescue by restoring MUC1-CD expression (Supplementary Fig. S2A–S2F). IRF1 was also necessary for STAT1 expression (Fig. 1G and H), indicating that MUC1-C regulates chromatin accessibility and activation of *STAT1* and *IRF1* in an auto-inductive circuit.

### MUC1-C induces PBRM1-mediated chromatin accessibility and expression of *IRF1* and *STAT1*

**Figure 2.**
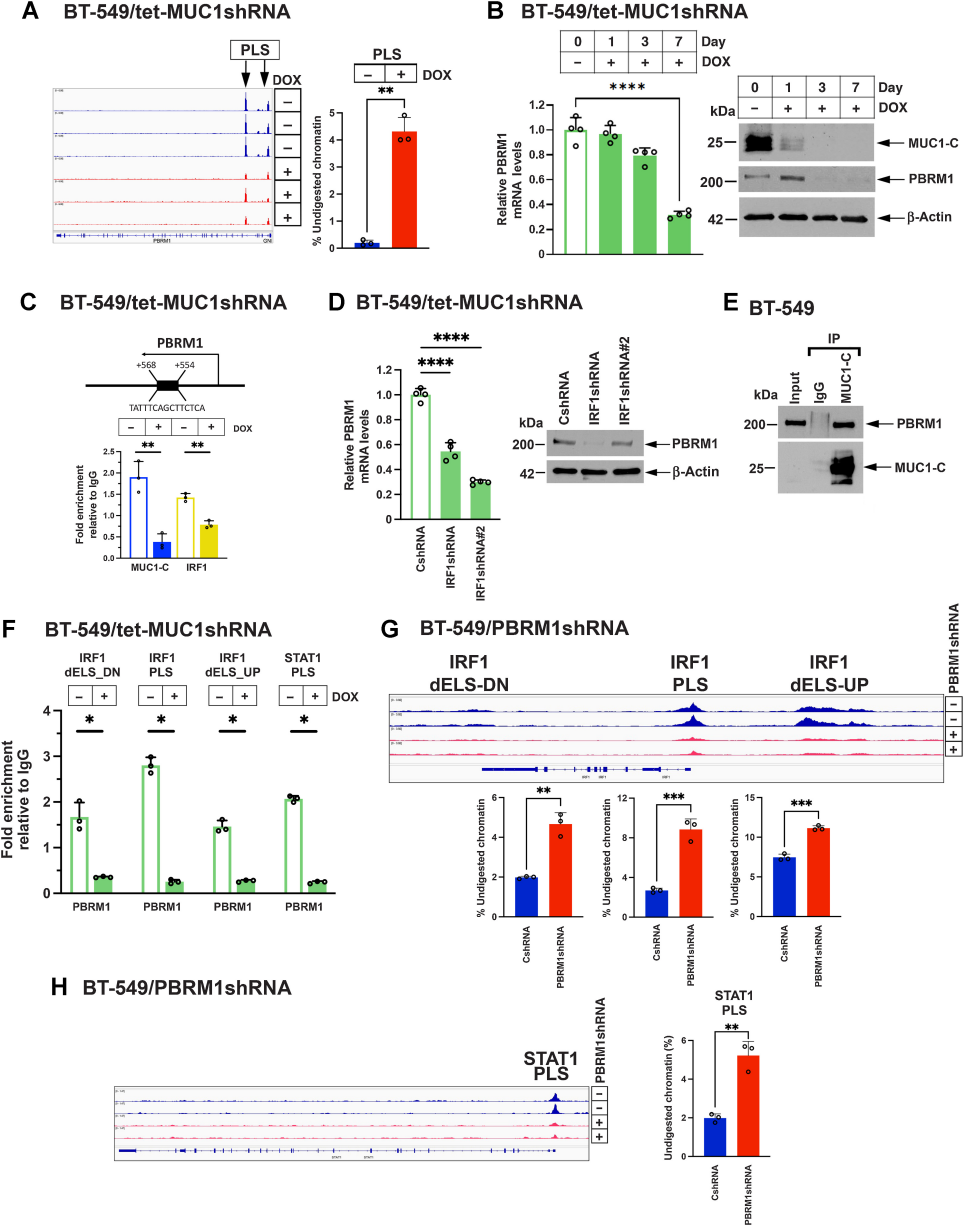
MUC1-C regulates PBRM1 expression and function in activating the *STAT1* and *IRF1* genes. **A,** Genome browser snapshots of ATAC-seq data from the *PBRM1* gene in BT-549/tet-MUC1shRNA cells treated with vehicle or DOX for 7 days (left). Chromatin was analyzed for accessibility by nuclease digestion (right). The results are expressed as the percentage of undigested chromatin (mean ± SD and individual values). **B,** BT-549/tet-MUC1shRNA cells treated with vehicle or DOX for the indicated days were analyzed for PBRM1 mRNA levels by qRT-PCR (left). The results (mean ± SD and individual values) are expressed as relative mRNA levels as compared with that obtained in control vehicle-treated cells (assigned a value of 1). Lysates were immunoblotted with antibodies against the indicated proteins (right). **C,** Schema of the *PBRM1* gene with localization of a PLS downstream to the TSS. Soluble chromatin from BT-549/tet-MUC1shRNA cells treated with vehicle or DOX for 7 days was precipitated with a control IgG, anti–MUC1-C and anti-IRF1. The DNA samples were amplified by qPCR with primers for the *PBRM1* PLS region. The results (mean ± SD and individual values) are expressed as relative fold enrichment as compared with that obtained with IgG (assigned a value of 1). **D,** BT-549/CshRNA, BT-549/IRF1shRNA and BT-549/IRF1shRNA#2 cells were analyzed for PBRM1 mRNA levels by qRT-PCR (left). The results (mean ± SD and individual values) are expressed as relative mRNA levels as compared with that obtained in control vehicle-treated cells (assigned a value of 1). Lysates were immunoblotted with antibodies against the indicated proteins (right). **E,** Nuclear lysates from BT-549 cells were precipitated with a control IgG and anti–MUC1-C. Input proteins and precipitates were immunoblotted with antibodies against the indicated proteins. **F,** Soluble chromatin from BT-549/tet-MUC1shRNA cells treated with vehicle or DOX for 7 days was precipitated with a control IgG and anti-PBRM1. The DNA samples were amplified by qPCR with primers for the indicated *IRF1* and *STAT1* regions. The results (mean ± SD and individual values) are expressed as fold-enrichment as compared with that obtained from control IgG-precipitated chromatin (assigned a value of 1). **G,** Genome browser snapshots of ATAC-seq data from the indicated *IRF1* regions in BT-549/CshRNA and BT-549/PBRM1shRNA cells. Chromatin from the indicated *IRF1* regions was analyzed for accessibility by nuclease digestion. The results are expressed as the percentage of undigested chromatin (mean ± SD and individual values). **H,** Genome browser snapshots of ATAC-seq data from the *STAT1* PLS in BT-549/CshRNA and BT-549/PBRM1shRNA cells. Chromatin was analyzed for accessibility by nuclease digestion. The results are expressed as the percentage of undigested chromatin (mean ± SD and individual values). *, *P* ≤ 0.05; **, *P* ≤ 0.01; ***, *P* ≤ 0.001; ****, *P* ≤ 0.0001.

In searching for chromatin remodeling complexes that contribute to *IRF1* and *STAT1* activation, we found that silencing MUC1-C is associated with loss of chromatin accessibility in the PBAF *PBRM1* gene (Fig. 2A) and suppression of PBRM1 expression (Fig. 2B, Supplementary Fig. S3A and S3B). In support of PBRM1 involvement in the MUC1-C/IRF1/STAT1 circuit, we found that MUC1-C and IRF1 occupied the *PBRM1* PLS (Fig. 2C), and that IRF1 is necessary for PBRM1 expression (Fig. 2D). Co-immunoprecipitation of nuclear lysates demonstrated that MUC1-C associates with PBRM1 (Fig. 2E). In addition, we found that, like MUC1-C, PBRM1 occupies the (i) *IRF1* PLS and dELSs and (ii) *STAT1* PLS, and that silencing MUC1-C decreases their occupancy (Fig. 2F). As shown for MUC1-C, silencing PBRM1 decreased chromatin accessibility of the (i) *IRF1* PLS and dELSs (Fig. 2G) and (ii) *STAT1* PLS (Fig. 2H), with downregulation of IRF1 and STAT1 expression (Supplementary Fig. S3C–S3F). These results support a MUC1-C→PBRM1 pathway, which activates *IRF1* and *STAT1* in an auto-inductive circuit.

### MUC1-C activates PBRM1-dependent activation of IRF1 target genes

**Figure 3.**
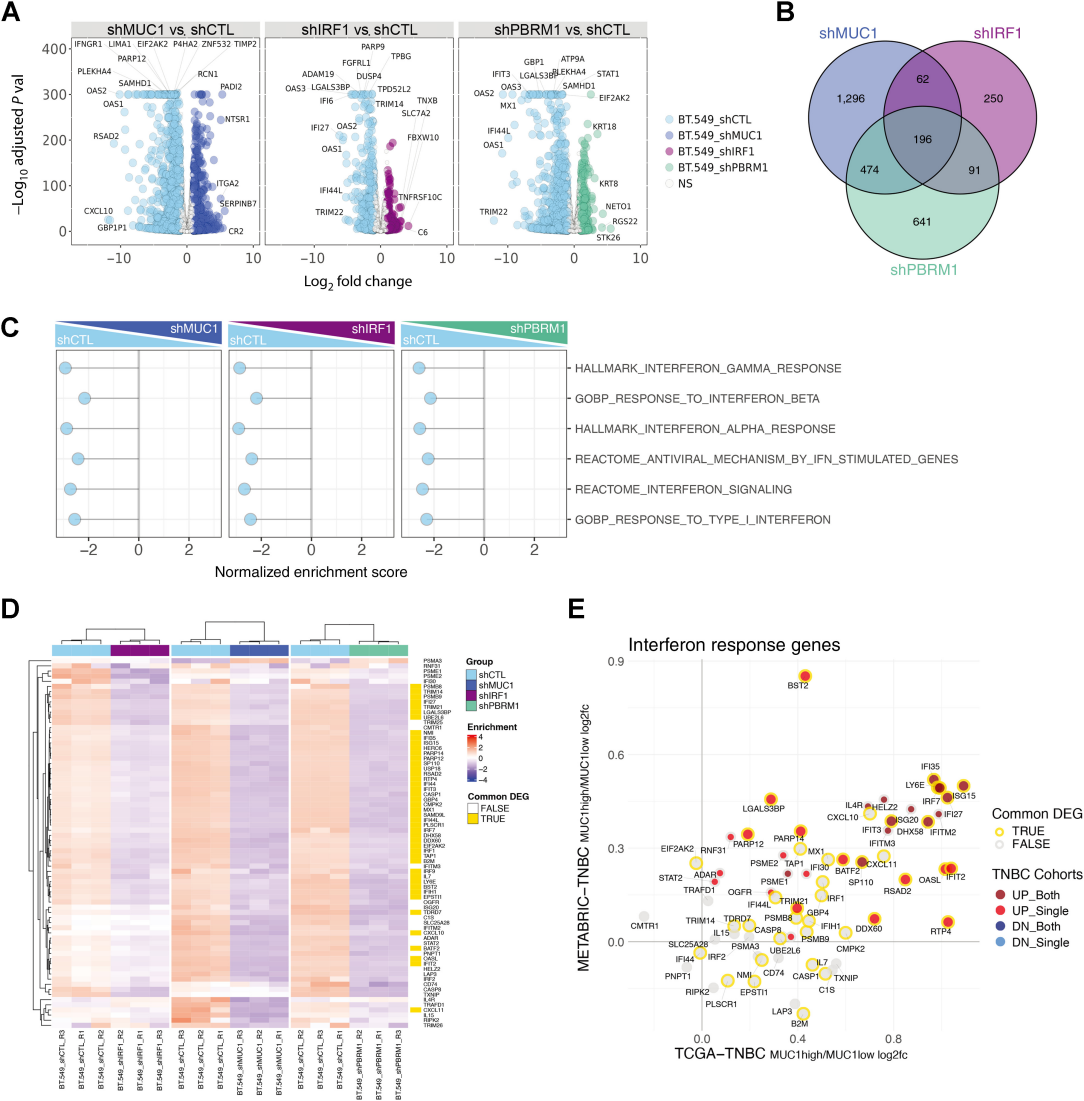
MUC1, PBRM1, and IRF1 regulate similar gene sets that are shared with TNBC tumors. **A,** RNA-seq was performed in triplicate on BT-549 cells silenced for MUC1, PBRM1, and IRF1. The datasets were analyzed for effects of MUC1-C silencing on repressed and activated genes as depicted by the Volcano plots. **B,** Venn diagram depicting the overlap of 196 downregulated genes in BT-549 cells silenced for MUC1, IRF1, and PBRM1 (Supplementary Table S3). **C,** RNA-seq datasets from BT-549 cells silenced for MUC1, IRF1, and PBRM1 were analyzed with GSEA for enrichment distribution using the indicated IFN-related gene signatures. **D,** Heatmaps of 72 common genes between the HALLMARK INTERFERON GAMMA RESPONSE and HALLMARK INTERFERON ALPHA RESPONSE signatures in BT-549 cells silenced for MUC1, IRF1, and PBRM1. The row indicator shows the 45 common genes from the 196 DEGs identified in B (yellow, Supplementary Table S4). **E,** Common up- and downregulated IFN response genes (detectable 66 genes out of 72 genes) in MUC1-high versus MUC1-low TNBC tumors from the METABRIC and TCGA-BRCA datasets. The outline (gold/gray) represents common DEGs in BT549 cells identified in D. The red/gray/blue dot indicates significantly up/downregulated genes (MUC1high vs. MUC1low) in one or both of the TCGA-BRCA/METABRIC cohorts.

IRF1 is essential for activation of the type I and II IFN pathways (8, 9). To determine whether MUC1-C and IRF1 are necessary for the activation of these genes, we found that, similar to STAT1, MUC1-C forms a nuclear complex with IRF1 (Supplementary Fig. S4A). MUC1-C includes 58 aa extracellular, 28 aa transmembrane, and 72 aa cytoplasmic domains (Supplementary Fig. S4B). *In vitro* studies demonstrated that the MUC1-C cytoplasmic 72 aa domain binds to IRF1 (Supplementary Fig. S4C, left) and that this interaction is conferred by IRF1-N (1–163), which includes the DNA-binding domain (Supplementary Fig. S4C, right). Further analysis demonstrated that (i) MUC1-CD (1–45), but not MUC1-CD (46–72; Supplementary Fig. S4D, left), and (ii) MUC1-CD CQC motif (Supplementary Fig. S4D, right) are necessary for binding to IRF1. On the basis of the interactions among MUC1-C, PBRM1, and IRF1, analysis of global transcriptional profiles (RNA-seq) of BT-549 cells demonstrated that MUC1-C, PBRM1, and IRF1 silencing results in broad changes in gene expression (956, 252, and 371 upregulated and 2028, 599, and 1402 downregulated genes in MUC1shRNA, IRF1shRNA, and PBRM1shRNA cells relative to controls, respectively; FDR<0.05, fold change (FC)>2; Fig. 3A), with 196 commonly downregulated (MUC1-C, PBRM1, and IRF1 induced) genes (Fig. 3B; Supplementary Table S3). Assessment of the top affected pathways by GSEA revealed strong associations of MUC1-C, PBRM1, and IRF1 with IFN-regulated gene sets (Fig. 3C). Consistent with these observations, we found that silencing MUC1-C, PBRM1, and IRF1 resulted in the downregulation of IFN pathway-responsive genes, including *IRF1* itself (Fig. 3D; Supplementary Table S4). Moreover, these common MUC1-, PBRM1-, and IRF1-activated genes were upregulated in MUC1-high versus MUC1-low tumors in the TNBC TCGA-BRCA and TNBC METABRIC cohorts (Fig. 3E). In support of these findings, analysis of RNA-seq data from BT-549 and MDA-MB-436 cells with MUC1-C silencing identified common sets of IRF1 target genes in the HALLMARK IFNA and IFNG RESPONSE signatures associated with immune evasion and DNA damage resistance (Supplementary Fig. S4E and S4F). In accordance with RNA-seq analysis, ATAC-seq data from MUC1-C silenced BT-549 cells revealed that many of the common MUC1-, PBRM1-, and IRF1-activated genes exhibit decreases in chromatin accessibility (Supplementary Fig. S4G).

### MUC1-C, PBRM1, and IRF1 signaling activate the type II IFN pathway *IDO1* and *WARS* genes

**Figure 4.**
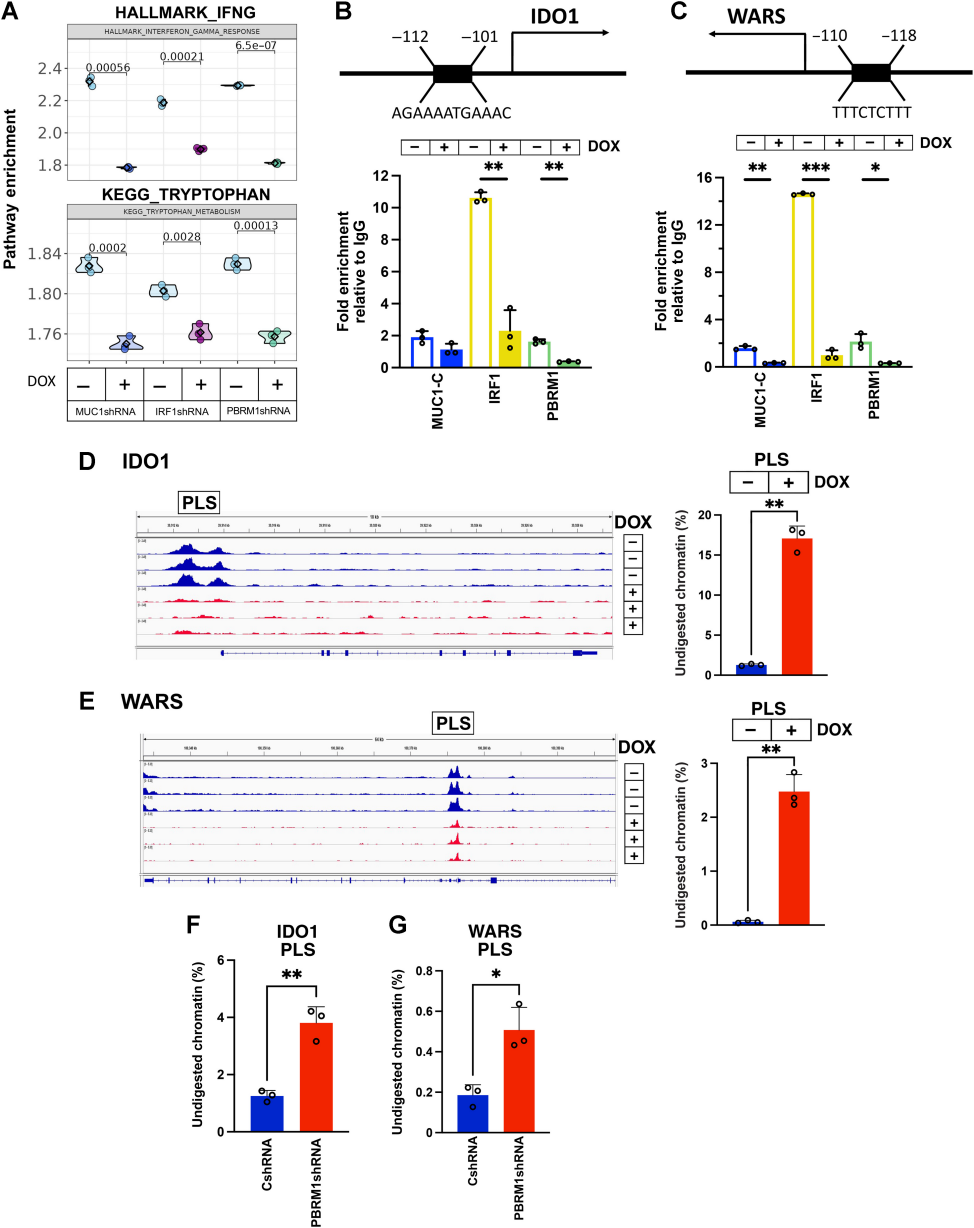
MUC1-C/PBRM1/IRF1 complexes induce chromatin accessibility and expression of the *IDO1* and *WARS* genes. **A,** Candidate pathway enrichment plot for the IFNG response and tryptophan metabolism in BT-549 cells silenced for MUC1-C, IRF1, and PBRM1. **B,** Schema of the *IDO1* gene with localization of a PLS upstream to the TSS. Soluble chromatin from BT-549/tet-MUC1shRNA cells treated with vehicle or DOX for 7 days was precipitated with a control IgG, anti-MUC1-C, anti-IRF1, and anti-PBRM1. The DNA samples were amplified by qPCR with primers for the *IDO1* PLS region. The results (mean ± SD and individual values) are expressed as relative fold enrichment as compared with that obtained with IgG (assigned a value of 1). **C,** Schema of the *WARS* gene with localization of a PLS upstream to the TSS. Soluble chromatin from BT-549/tet-MUC1shRNA cells treated with vehicle or DOX for 7 days was precipitated with a control IgG, anti–MUC1-C, anti-IRF1, and anti-PBRM1. The DNA samples were amplified by qPCR with primers for the *WARS* PLS region. The results (mean ± SD and individual values) are expressed as relative fold enrichment as compared with that obtained with IgG (assigned a value of 1). **D** and **E,** Genome browser snapshots of ATAC-seq data from the *IDO1* (**D**) and *WARS* (**E**) PLS regions in BT-549/tet-MUC1shRNA cells treated with vehicle or DOX for 7 days (left). Chromatin was analyzed for accessibility by nuclease digestion (right). The results are expressed as the percentage of undigested chromatin (mean ± SD and individual values). **F** and **G,** Chromatin from BT-549/CshRNA and BT-549/PBRM1shRNA cells was analyzed for accessibility of the *IDO1* (**F**) and *WARS* (**G**) PLS regions by nuclease digestion. The results are expressed as the percentage of undigested chromatin (mean±SD and individual values). *, *P* ≤ 0.05; **, *P* ≤ 0.01; ***, *P* ≤ 0.001.

GSEA and GSVA revealed that MUC1-C, PBRM1, and IRF1 silencing in BT-549 cells is associated with marked decreases in both type II IFN pathway and tryptophan-related pathway enrichment (Fig. 4A). Analysis of the ATAC-seq data further demonstrated that silencing MUC1 decreases chromatin accessibility of genes encoding effectors of the type II IFN pathway (Supplementary Fig. S5A). Among these, we identified *IDO1*, which encodes indoleamine-2,3-dioxygenase-1. IDO1 reduces tryptophan (Trp) levels in the tumor microenvironment (TME) that are necessary for T cell proliferation and function (50). Transcriptional profiles of BT-549 cells silenced for MUC1-C, PBRM1, and IRF1 showed a marked suppression of IDO1 expression (Supplementary Fig. S5B). In addition, we found suppression of tryptophanyl-tRNA synthetase (WARS, WRS), which protects cancer cells from Trp depletion (Supplementary Fig. S5B; ref. 50). Further analysis of BT-549 cells silenced for MUC1-C and PBRM1 demonstrated downregulation of IDO1 and WARS (Supplementary Fig. S2E and S2F; Supplementary Fig. S5C). Accordingly, we investigated whether MUC1-C, PBRM1, and IRF1 are necessary for activation of the *IDO1* and *WARS* genes. The *IDO1* promoter region includes an IRF1/ISRE binding motif upstream of the TSS (Fig. 4B). Consistent with the demonstration that MUC1-C forms a complex with PBRM1 and IRF1, we found that MUC1-C, PBRM1, and IRF1 occupy this region (Fig. 4B). The *WARS* promoter region also contains an ISRE (Fig. 4C). Similar to *IDO1*, we found that the *WARS* ISRE is occupied by MUC1-C, PBRM1, and IRF1 (Fig. 4C), and that silencing MUC1-C (Supplementary Fig. S6A, left and right), IRF1 (Supplementary Fig. S6B, left and right), and PBRM1 (Supplementary Fig. S6C, left and right) downregulates IDO1 and WARS expression. In MDA-MB-436 cells, which have undetectable IDO1 expression, silencing MUC1-C (Supplementary Fig. S6D, left and right), IRF1 (Supplementary Fig. S6E, left and right), and PBRM1 (Supplementary Fig. S6F, left and right) decreased WARS transcript and protein levels. As observed for *STAT1* and *IRF1*, MUC1-C was necessary for increases in chromatin accessibility in the *IDO1* PLS (Fig. 4D) and *WARS* PLS (Fig. 4E) regions. Moreover, PBRM1 was necessary for opening chromatin at the *IDO1* PLS (Fig. 4F) and *WARS* PLS (Fig. 4G), supporting a MUC1-C pathway that involves PBRM1 and IRF1 in driving IDO1 and WARS expression.

### MUC1-C, PBRM1, and IRF1 are necessary for activation of the type I IFN pathway *RIG-I, MDA5*, and *ISG15* genes

**Figure 5.**
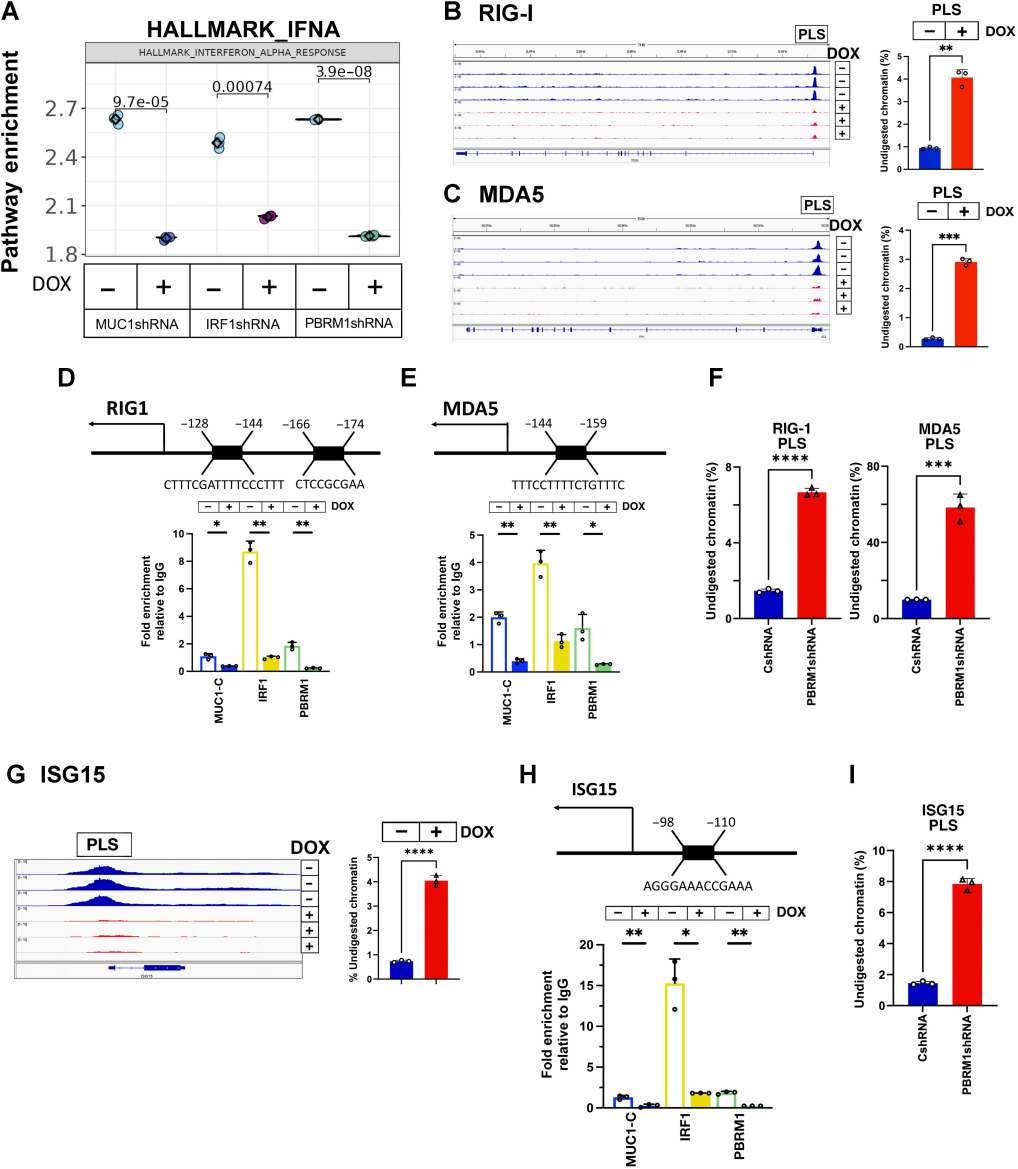
MUC1-C/PBRM1/IRF1 complexes induce chromatin accessibility and expression of the *RIG-I, MDA5*, and *ISG15* genes. **A,** Candidate pathway enrichment plot for the IFNA response in BT-549 cells silenced for MUC1-C, IRF1, and PBRM1. **B** and **C,** Genome browser snapshots of ATAC-seq data from the *RIG-I* (**B**) and *MDA5* (**C**) PLSs in BT-549/tet-MUC1shRNA cells treated with vehicle or DOX for 7 days (left). Chromatin was analyzed for accessibility by nuclease digestion (right). The results are expressed as the percentage of undigested chromatin (mean ± SD and individual values). **D** and **E,** Schema of the *RIG-I* (**D**) *and MDA5* (**E**) genes with localization of a PLS upstream to the TSS. Soluble chromatin from BT-549/tet-MUC1shRNA cells treated with vehicle or DOX for 7 days was precipitated with a control IgG, anti–MUC1-C, anti-IRF1 and anti-PBRM1. The DNA samples were amplified by qPCR with primers for the *RIG-I* (**D**) and *MDA5* (**E**) PLS regions. The results (mean ± SD and individual values) are expressed as relative fold enrichment as compared with that obtained with IgG (assigned a value of 1). **F,** Chromatin from BT-549/CshRNA and BT-549/PBRM1shRNA cells was analyzed for accessibility of the *RIG-I* and *MDA5* PLS regions by nuclease digestion. The results are expressed as the percentage of undigested chromatin (mean ± SD and individual values). **G,** Genome browser snapshots of ATAC-seq data from the *ISG15* PLS in BT-549/tet-MUC1shRNA cells treated with vehicle or DOX for 7 days (left). Chromatin was analyzed for accessibility by nuclease digestion (right). The results are expressed as the percentage of undigested chromatin (mean ± SD and individual values). **H,** Schema of the *ISG15* gene with localization of a PLS upstream to the TSS. Soluble chromatin from BT-549/tet-MUC1shRNA cells treated with vehicle or DOX for 7 days was precipitated with a control IgG, anti-MUC1-C, anti-IRF1, and anti-PBRM1. The DNA samples were amplified by qPCR with primers for the *ISG15* PLS region. The results (mean ± SD and individual values) are expressed as relative fold enrichment as compared with that obtained with IgG (assigned a value of 1). **I,** Chromatin from BT-549/CshRNA and BT-549/PBRM1shRNA cells was analyzed for accessibility of the *ISG15* PLS region by nuclease digestion. The results are expressed as the percentage of undigested chromatin (mean ± SD and individual values). *, *P* ≤ 0.05; **, *P* ≤ 0.01; ***, *P* ≤ 0.001; ****, *P* ≤ 0.0001.

MUC1-C, PBRM1, and IRF1 silencing in BT-549 cells was also associated with a marked decrease in enrichment of the type I IFN pathway (Fig. 5A). Moreover, ATAC-seq data demonstrated that silencing MUC1-C decreases the chromatin accessibility of genes in the type I IFN pathway (Supplementary Fig. S7A), which is activated in part by the RIG-I and MDA5 cytosolic RNA-sensing PRRs (51). As shown for *IDO1* and *WARS*, we identified a PLS in the *RIG-I* gene and found that silencing MUC1-C suppresses chromatin accessibility in this region (Fig. 5B). In addition, we found that (i) *MDA5* contains a PLS, which is also dependent on MUC1-C for opening chromatin (Fig. 5C), (ii) the *RIG-I* PLS is occupied by MUC1-C, PBRM1 and IRF1, and (iii) silencing MUC1-C decreases their occupancy (Fig. 5D). Similar results were obtained for *MDA5* PLS (Fig. 5E). Consistently, silencing PBRM1 decreased chromatin accessibility in the *RIG-I* and *MDA5* PLS regions (Fig. 5F). Moreover, silencing of MUC1-C, PBRM1, and IRF1 downregulated RIG-I and MDA5 expression (Supplementary Fig. S2E and S2F; Supplementary Fig. S7B–S7S7G). Chronic activation of the type I IFN pathway by low levels of IFNβ production in cancer cells has been linked to induction of the IRDS (4, 5). Analysis of genes in the IRDS demonstrated that MUC1-C silencing is associated with decreases in chromatin accessibility (Supplementary Fig. S8A). These results supported the involvement of MUC1-C–induced chromatin remodeling in integrating type I and II IFN inflammatory pathways with DNA damage resistance. Among the MUC1-induced IRDS genes, we identified *ISG15*, which is overexpressed in human cancers and couples chronic inflammation with DNA damage resistance (52). Silencing MUC1-C was associated with a marked decrease in chromatin accessibility within a broad *ISG15* PLS region (Fig. 5G). We found that the *ISG15* PLS is occupied by MUC1-C, PBRM1, and IRF1, and that silencing MUC1-C decreases their occupancy (Fig. 5H). Silencing PBRM1 also decreased chromatin accessibility in the *ISG15* PLS region (Fig. 5I). Moreover, silencing MUC1-C, PBRM1, and IRF1 downregulated ISG15 expression in BT-549 and MDA-MB-436 cells (Supplementary Fig. S2E and S2F; Supplementary Fig. S8B–S8S8G).

### Targeting MUC1-C inhibits DNA damage resistance in TNBC CSCs

**Figure 6.**
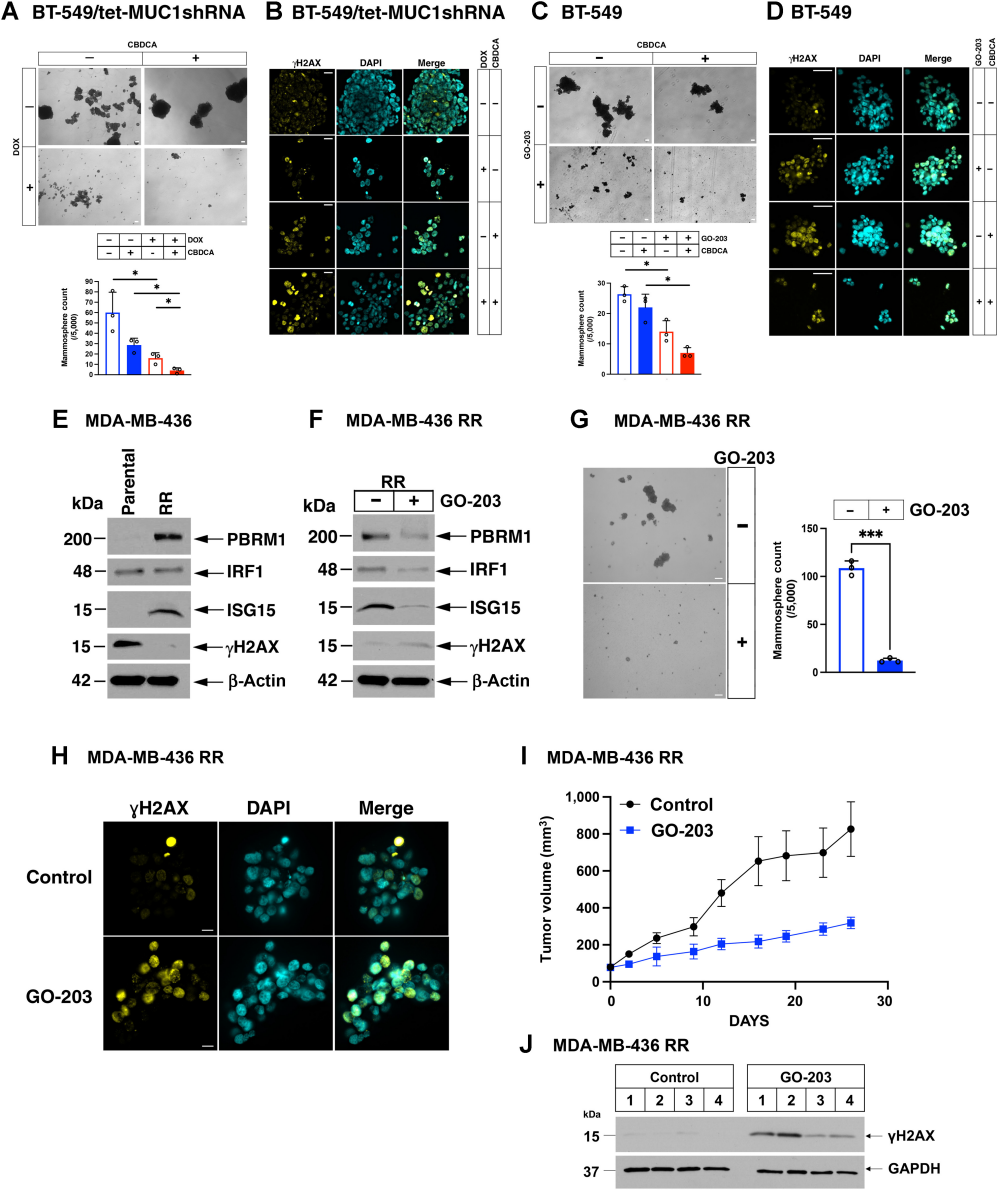
Targeting MUC1-C in TNBC CSCs inhibits self-renewal capacity and circumvents DNA damage resistance. **A,** BT-549/tet-MUC1shRNA cells were treated with vehicle or DOX for 7 days and then assayed for tumorsphere formation in the absence and presence of 0.2 μmol/L CBDCA for 7 days (**A,** top); scale bar, 100 μm. The results (mean ± SD of 3 biological replicates) are expressed as the number of mammospheres (**A,** lower). **B,** BT-549/tet-MUC1shRNA mammospheres treated with vehicle or DOX in the absence and presence of 2 μmol/L CBDCA for 2 days were assayed by ICC analysis for visualization of γH2AX foci; scale bar, 50 μm. **C,** BT-549 cells treated with vehicle or 0.75 μmol/L GO-203 in the absence and presence of 0.2 μmol/L CBDCA were assayed for mammosphere formation at 8 days (top); scale bar, 100 μm. The results (mean ± SD of 3 biological replicates) are expressed as the number of mammospheres (bottom).**D,** BT-549 mammospheres treated with vehicle or 2.5 μmol/L GO-203 in the absence and presence of 2 μmol/L CBDCA for 2 days were assayed by ICC analysis for visualization of γH2AX foci; scale bar, 50 μm. **E,** Lysates from parental MDA-MB-436 and olaparib-resistant MDA-MB-436RR cells were immunoblotted with antibodies against the indicated proteins. **F,** MDA-MB-436RR cells treated with 5 μmol/L GO-203 for 2 days were immunoblotted with antibodies against the indicated proteins. **G,** MDA-MB-436RR cells treated with vehicle or 5 μmol/L GO-203 were assayed for mammosphere formation at 7 days (left); scale bar, 100 μm. The results (mean±SD of 3 biological replicates) are expressed as the number of mammospheres (right). **H,** MDA-MB-436RR mammospheres treated with vehicle or 2.5 μmol/L GO-203 for 2 days were assayed by ICC analysis for visualization of γH2AX foci; scale bar, 50 μm. **I,** Six-week-old nude mice were injected subcutaneously in the flank with 3×10^6^ MDA-MB-436RR cells. Mice pair-matched into two groups when tumors reached 100 to 150 mm^3^ were treated intraperitoneally daily with PBS or GO-203 for 26 days. Tumor volumes are expressed as the mean±SEM for 6 mice. **J,** Lysates from tumors exposed for 5 days to GO-203 were immunoblotted with antibodies against the indicated proteins. *, *P* ≤ 0.05; ***, *P* ≤ 0.001.

The CSC state is associated with DNA damage resistance (53). MUC1-C induces the TNBC CSC state; however, it is unknown whether MUC1-C drives resistance to DNA damage in TNBC CSCs (24, 31, 32, 54, 55). To determine whether MUC1-C plays a role in the emergence of persister cells under the stress of DNA-damaging agents, we established BT-549/tet-MUC1shRNA TNBC CSCs based on their capacity for self-renewal in forming mammospheres (Fig. 6A). Silencing MUC1-C with DOX treatment decreased mammosphere formation (Fig. 6A). We also found that silencing MUC1-C in these CSCs significantly reduces their capacity for self-renewal when combined with carboplatin (CBDCA) treatment (Fig. 6A). This loss of self-renewal was associated with induction of DNA damage, as evidenced by the formation of γH2AX foci (Fig. 6B). Silencing PBRM1 and IRF1 also suppressed self-renewal (Supplementary Fig. S9A and S9B), confirming that MUC1-C→PBRM1→IRF1 signaling contributes to the CSC state. As an additional approach, we treated BT-549 cells with the GO-203 inhibitor, which is a cell-penetrating peptide that blocks MUC1-C homodimerization, nuclear localization, and function (24). Administration of GO-203 in mice and humans has achieved plasma levels of approximately 2 μmol/L in the absence of dose-limiting toxicity (24). Treatment of BT-549 CSCs with 0.75 μmol/L GO-203 and carboplatin showed greater inhibition of self-renewal than treatment with either agent alone (Fig. 6C) in association with the induction of γH2AX foci (Fig. 6D). Platinum-based therapies remain the standard of care for advanced BRCA wild-type TNBCs; whereas PARP inhibitors are used for the treatment of BRCA mutant disease (56). In studies of BRCA mutant MDA-MB-436 cells, we found that GO-203 also potentiates the effects of olaparib on loss of self-renewal capacity (Supplementary Fig. S9C) and overcoming DNA damage resistance (Supplementary Fig. S9D), indicating that MUC1-C protects TNBC CSCs from treatment with genotoxic anticancer drugs. Given these findings, we next investigated whether MUC1-C is important for acquired resistance to DNA-damaging agents. To this end, we analyzed MDA-MB-436 cells established for resistance to rucaparib, which exhibit cross-resistance to olaparib and cisplatin (Supplementary Fig. S9E; refs. 35, 57). We found that resistant MDA-MB-436RR cells have increased expression of PBRM1 and ISG15 (Fig. 6E). Moreover, treatment with GO-203 was associated with (i) downregulation of PBRM1, IRF1 and ISG15, and (ii) induction of γH2AX (Fig. 6F). GO-203 treatment of MDA-MB-436RR cells inhibited cell viability (Supplementary Fig. S9F), resulted in a pronounced decrease in self-renewal capacity (Fig. 6G) and reversed DNA damage resistance, as evidenced by increases in γH2AX foci (Fig. 6H). In extending this analysis to established MDA-MB-436RR tumors in mice, we found that GO-203 treatment is effective in inhibiting their growth (Fig. 6I) in association with induction of DNA damage, as evidenced by increases in γH2AX expression (Fig. 6J). Collectively, these findings demonstrate that targeting MUC1-C with GO-203 circumvents the intrinsic and acquired DNA damage resistance of TNBC CSCs and supports combining GO-203 with carboplatin or olaparib for the treatment of recalcitrant TNBCs.

### Involvement of MUC1-C in conferring DNA damage resistance and immune evasion in TNBC

**Figure 7.**
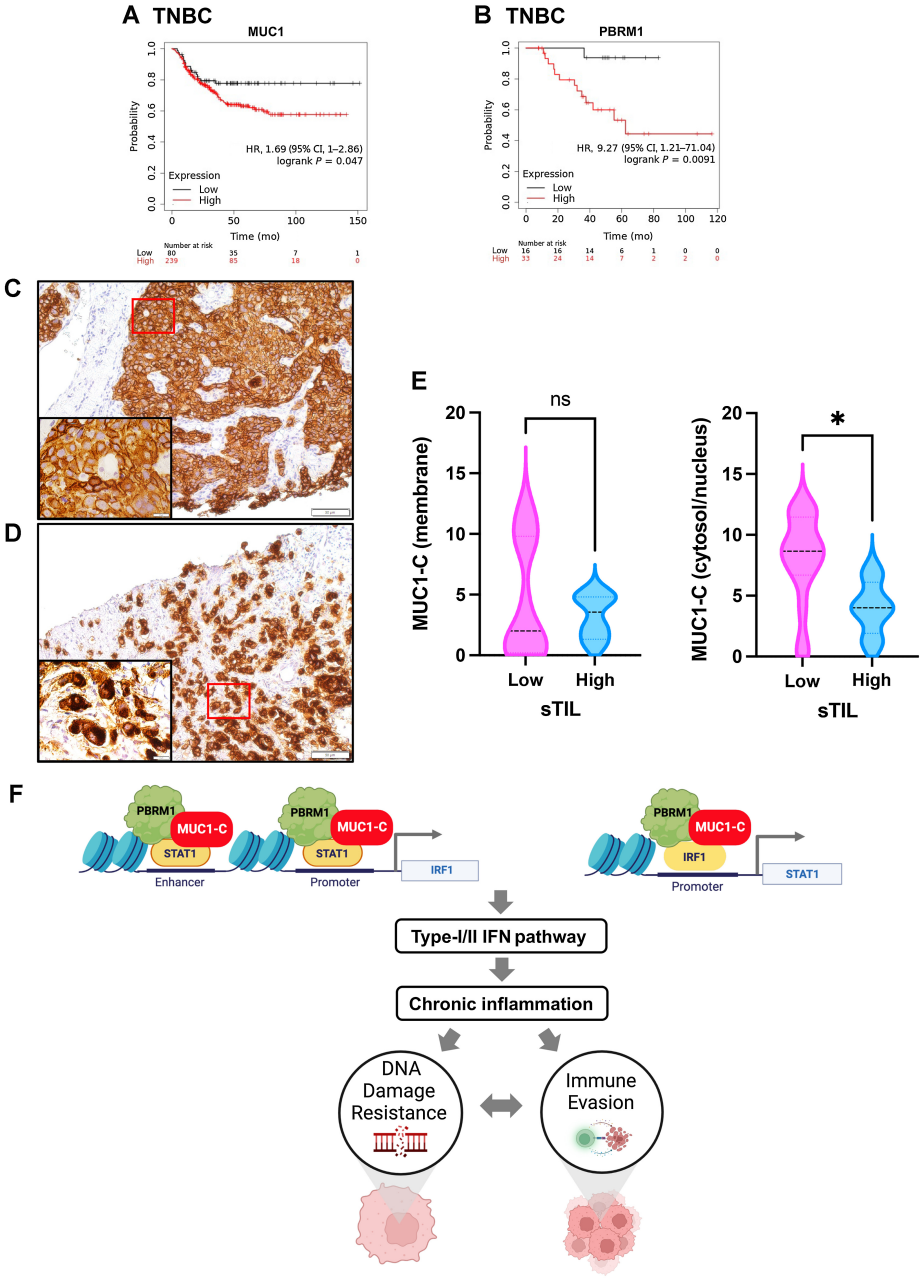
MUC1 and PBRM1 associate with decreases in responsiveness of TNBC tumors to chemotherapy. **A** and **B,** Kaplan–Meier curves for relapse-free survival created by the public database and web application KM plotter (http://kmplot.com/ analysis/) based on the MUC1 (**A**) and PBRM1 (**B**) expression levels. Patients with TNBC were stratified with high (red) or low (black) expression of MUC1 and PBRM1. **C** and **D,** Representative MUC1-C staining in cell membrane (**C**) and cytosol/nuclear (**D**) of primary TNBC samples (magnifications, ×20). Insets highlight localization of MUC1-C expression in cancer cells (magnifications, ×100). **E,** TNBC tumors with cell membrane (left) and cytosol/nuclear (right) MUC1-C expression were stratified with sTIL levels in TNBC samples. **F,** Proposed model based on the present data demonstrating that MUC1-C induces PBRM1 expression and, in turn, binds to PBRM1. MUC1-C/PBRM1 complexes associate with (i) STAT1 in activating the *IRF1* gene and (ii) IRF1 in activating *STAT1* by increasing chromatin accessibility and their transcription. Induction of STAT1 and IRF1 expression contributes to a feed-forward circuit in which MUC1-C/PBRM1/STAT1 and MUC1-C/PBRM1/IRF1 complexes drive activation of genes in the type I and II IFN pathways. In this way, prolonged MUC1-C activation in settings of repetitive cycles of damage and repair promote chronic inflammation. A consequence of persistent MUC1-C activation and PBRM1-mediated chromatin remodeling is the establishment of IFN gene signatures that contribute to the CSC state and DNA damage resistance. DNA damage-induced inflammatory signaling is coupled to immune evasion, which in turn contributes to a MUC1-C/PBRM1-driven auto-inductive circuit that integrates a refractory state to treatment with genotoxic and immunotherapeutic agents. Figure created with BioRender.com. *, *P* ≤ 0.05.

Analysis of scRNA-seq datasets has demonstrated that MUC1 is widely expressed in TNBC tumor cell populations (36). However, it is not known whether MUC1 is associated with adverse outcomes in patients with TNBC treated with genotoxic agents. On the basis of the present findings that MUC1-C is important for drug resistance, we analyzed MUC1 expression and clinical response in a cohort of patients with TNBC treated with cytotoxic anticancer agents. We found that patients with MUC1-high versus MUC1-low tumors experienced poor clinical outcomes (Fig. 7A). We also found that patients with PBRM1-high versus PBRM1-low tumors had significantly decreased relapse-free survival (Fig. 7B), supporting the involvement of the MUC1-C→PBRM1 pathway in conferring DNA damage resistance. An important factor in the responsiveness of TNBCs to chemotherapy is the presence of TILs (58, 59). In this respect, patients with TNBCs harboring high TILs in the TME experience significant improvement in disease-free survival in response to chemotherapy compared with those with low TILs (58, 59). Expression of MUC1 transcripts in TNBCs is associated with the depletion of TILs in the TME (36); however, there have been no previous studies examining MUC1-C expression in TNBCs or the relationship between MUC1-C and the presence of TILs. Accordingly, we studied MUC1-C expression by IHC analysis of TNBC core biopsies and found distinct patterns of cell membrane (Fig. 7C) and cytosolic/nuclear (Fig. 7D) staining. These contrasting expression patterns correspond to the internalization of MUC1-C from the cell membrane to the cytoplasm and nucleus during the progression of TNBC cells to the CSC state (32). Further analysis demonstrated that TNBC tumors with MUC1-C membrane staining are not associated with TMEs harboring significant differences in stromal TILs (sTILs; Fig. 7E). In contrast, TNBC tumors with MUC1-C cytosolic/nuclear staining were significantly associated with decreased sTILs levels (Fig. 7E). These findings indicated that TNBC progression contributes to TIL depletion, which is an adverse factor for responsiveness to genotoxic anticancer agents.

## Discussion

PBRM1 represses, as well as activates, IFN pathway genes in different cancer cells (18, 21, 22). PBRM1 status has also been associated with responsiveness to ICIs in selected cancers and not others (18–23). The basis for these disparate outcomes is unclear, emphasizing the need for a better understanding of how PBRM1 regulates IFN pathway genes in the malignant setting. The present results demonstrate that MUC1-C induces PBRM1 in TNBC cells and, in turn, forms a nuclear complex with PBRM1 (Fig. 7F). MUC1-C chronically activates the type II IFN pathway and downstream immunosuppressive IDO1 and COX2 effectors in association with immune cell-depleted TMEs (36). MUC1-C has also been linked to induction of cytosolic nucleotide PRRs and STING in driving activation of the type I IFN pathway (41). These findings and the demonstration that MUC1-C interacts with PBRM1 suggested that MUC1-C may play a role in regulating PBRM1-mediated chromatin accessibility and the expression of ISGs. Consistent with this notion, we found that MUC1-C/STAT1 complexes recruit PBRM1 to *IRF1* PLS and ELS regions. In support of a functional MUC1-C, STAT1, and PBRM1 interaction, silencing MUC1-C decreased STAT1 and PBRM1 occupancy. MUC1-C and PBRM1 were also found to be necessary for (i) opening chromatin in the *IRF1* PLS and ELS regions and (ii) inducing IRF1 expression. Our results demonstrate that MUC1-C also directly interacts with IRF1 and that silencing MUC1-C decreases occupancy of PBRM1 and IRF1 on *STAT1* PLS. Moreover, silencing MUC1-C and PBRM1 decreased chromatin accessibility of the *STAT1* PLS, supporting a chronic MUC1-C–driven STAT1/IRF1/PBRM1 auto-inductive signaling network.

Our results extend this interaction between MUC1-C and PBRM1 to the activation of additional IRF1 target genes. As shown for *STAT1*, MUC1-C/PBRM1/IRF1 complexes induce chromatin accessibility and expression of the type II IFN pathway *IDO1* and *WARS* genes. In support of a common mechanism for the induction of IRF1 target genes, we also found that MUC1-C/PBRM1/IRF1 complexes are necessary for the induction of chromatin accessibility and the expression of the type I IFN pathway *RIG-I* and *MDA5* genes. These findings and the demonstration that silencing MUC1-C decreases chromatin accessibility and the expression of multiple other ISGs uncovered the involvement of MUC1-C and PBRM1 in chronic activation of type I and II IFN pathways (Fig. 7F). PBRM1 has pleiotropic functions that intersect with DNA damage repair (60). PBRM1 deficiency induces replication stress and confers synthetic lethality to DNA repair inhibitors (15). Replication stress potentiates the antitumor immune response by increasing genomic instability and activation of the STING pathway (16). Along these lines, we found that ISG15 expression is dependent on MUC1-C/PBRM1/IRF1 and increases in chromatin accessibility within the *ISG15* PLS. Of importance in this regard, ISG15 is overexpressed in cancer cells through unknown mechanisms (52, 61) and functions as a major effector of innate immunity, linking DNA damage resistance to immune evasion (Fig. 7F; ref. 62).

The CSC state is associated with DNA damage resistance and immune evasion (33, 34, 63). Our results demonstrate that MUC1-C/PBRM1/IRF1 signaling promotes the TNBC CSC state, as evidenced by dependence on their expression for self-renewal capacity. In parallel, MUC1-C recruits ARID1A/BAF to PLS and ELS regions of stemness genes in association with driving increases in chromatin accessibility and expression (31). These findings collectively support the involvement of MUC1-C and SWI/SNF-mediated chromatin remodeling in integrating chronic activation of the type I and II IFN pathways with the CSC state and DNA damage resistance (Fig. 7F). Platinum-based agents are often used for the treatment of TNBCs without germline *BRCA* mutations; however, their effectiveness is limited by intrinsic and acquired DNA damage resistance (56). Of potential translational relevance, we found that targeting MUC1-C in TNBC CSCs potentiates the effects of carboplatin by circumventing DNA damage resistance and promoting loss of self-renewal capacity. *BRCA* mutant TNBCs also exhibit primary and secondary resistance to PARP inhibitors (56). In this context, targeting MUC1-C in *BRCA*-mutant TNBC cells with the GO-203 inhibitor suppressed (i) PBRM1, IRF1, and ISG15 expression, (ii) self-renewal capacity, and (iii) DNA damage resistance. Similar results were obtained with GO-203 treatment of olaparib-resistant TNBC cells, confirming that MUC1-C is a target for suppressing the CSC state and resistance to genotoxic agents.

In extending these findings to TNBC tumors, we found that MUC1 and PBRM1 are associated with decreased RFS in patients treated with chemotherapy. We also found that MUC1-C expression in TNBCs is associated with depletion of sTILs, which decreases responsiveness to chemotherapy and survival (58, 59). These findings in TNBC tumors corroborate the involvement of MUC1-C at the intersection between DNA damage resistance and immune evasion (Fig. 7F). In summary, MUC1-C integrates PBRM1-mediated chromatin remodeling in driving chronic activation of type I and II IFN pathway genes, which are important for the CSC state and resistance to genotoxic and immunotherapeutic agents (Fig. 7F; ref. 64).

## Supplementary Material

Supplementary Figures S1-S9 and Tables S1-S4. S1. Silencing MUC1-C downregulates IRF1 expression in TNBC cells. S2. Effects of silencing MUC1-C on TNBC cells. S3. MUC1-C is necessary for PBRM1 expression A and B. S4. MUC1-C forms a direct complex with IRF1 that activates type I and II ISGs. S5. Common genes regulated in cells silenced for MUC1, IRF1, and PBRM1. S6. Silencing MUC1-C, IRF1, and PBRM1 downregulated IDO1 and WARS in TNBC cells. S7. Silencing of MUC1-C, IRF1, and PBRM1 downregulates RIG-I and MDA5 in TNBC cells. S8. Silencing MUC1-C, IRF1, and PBRM1 downregulates ISG15 expression in TNBC cells. S9. Effects of silencing IRF1 and PBRM1 on BT-549 mammosphere formation and GO-203 treatment on the viability of olaparib-resistant MDA-MB-436RR cells. Table S1. Primers used for qRT-PCR. Table S2. Primers used for ChIP-qPCR and DNase I chromatin accessibility assays. Table S3. Common downregulated 196 DEGs in BT-549 cells with silencing of MUC1, IRF1, and PBRM1. Table S4. Overlapping IFN-pathway genes downregulated in cells with MUC1-C, PBRM1, and IRF1 silencing.
