# Supplementary material for: MUC1-C Dictates PBRM1-Mediated Chronic Induction of Interferon Signaling, DNA Damage Resistance, and Immunosuppression in Triple-Negative Breast Cancer
Source: Mol Cancer Res. 2022 Nov 29;21(3):274–89. doi: 10.1158/1541-7786.MCR-22-0772 (PMC9975675; doi:10.1158/1541-7786.MCR-22-0772)
Supplement: Supplementary Figures S1-S9 and Tables S1-S4. — S1. Silencing MUC1-C downregulates IRF1 expression in TNBC cells. S2. Effects of silencing MUC1-C on TNBC cells. S3. MUC1-C is necessary for PBRM1 expression A and B. S4. MUC1-C forms a direct complex with IRF1 that activates type I and II ISGs. S5. Common genes regulated in cells silenced for MUC1, IRF1, and PBRM1. S6. Silencing MUC1-C, IRF1, and PBRM1 downregulated IDO1 and WARS in TNBC cells. S7. Silencing of MUC1-C, IRF1, and PBRM1 downregulates RIG-I and MDA5 in TNBC cells. S8. Silencing MUC1-C, IRF1, and PBRM1 downregulates ISG15 expression in TNBC cells. S9. Effects of silencing IRF1 and PBRM1 on BT-549 mammosphere formation and GO-203 treatment on the viability of olaparib-resistant MDA-MB-436RR cells. Table S1. Primers used for qRT-PCR. Table S2. Primers used for ChIP-qPCR and DNase I chromatin accessibility assays. Table S3. Common downregulated 196 DEGs in BT-549 cells with silencing of MUC1, IRF1, and PBRM1. Table S4. Overlapping IFN-pathway genes downregulated in cells with MUC1-C, PBRM1, and IRF1 silencing. [file mcr-22-0772_supplementary_figures_s1-s9_and_tables_s1-s4.suppsd1.pdf]

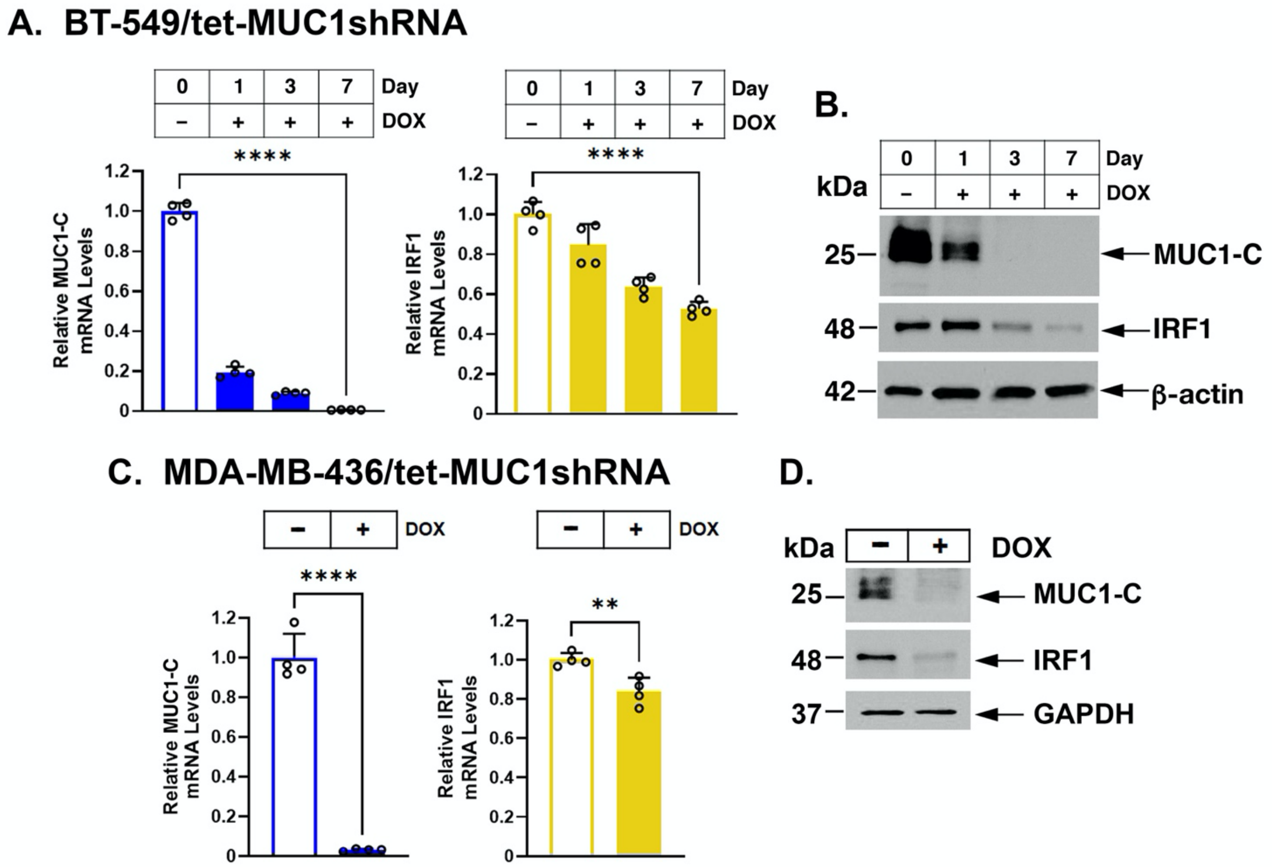

Supplemental Figure S1. Silencing MUC1-C downregulates IRF1 expression in TNBC cells. A and B. BT-549/tet-MUC1shRNA cells treated with vehicle or DOX for 7 days were analyzed for MUC1-C and IRF1 mRNA levels using qRT-PCR (**A**). The results (mean±SD and individual values) are expressed as relative mRNA levels compared to those obtained in the control vehicle-treated cells (assigned a value of 1). Lysates were immunoblotted using antibodies against the indicated proteins (**B**). **C and D.** MDA-MB-436/tet-MUC1shRNA cells treated with DOX vehicle for 7 days were analyzed for MUC1 and IRF1 mRNA levels by qRT-PCR (**C**). The results (mean ± SD and individual values) are expressed as relative mRNA levels compared to those obtained in control vehicle-treated cells (assigned a value of 1). Lysates were immunoblotted using antibodies against the indicated proteins (**D**).

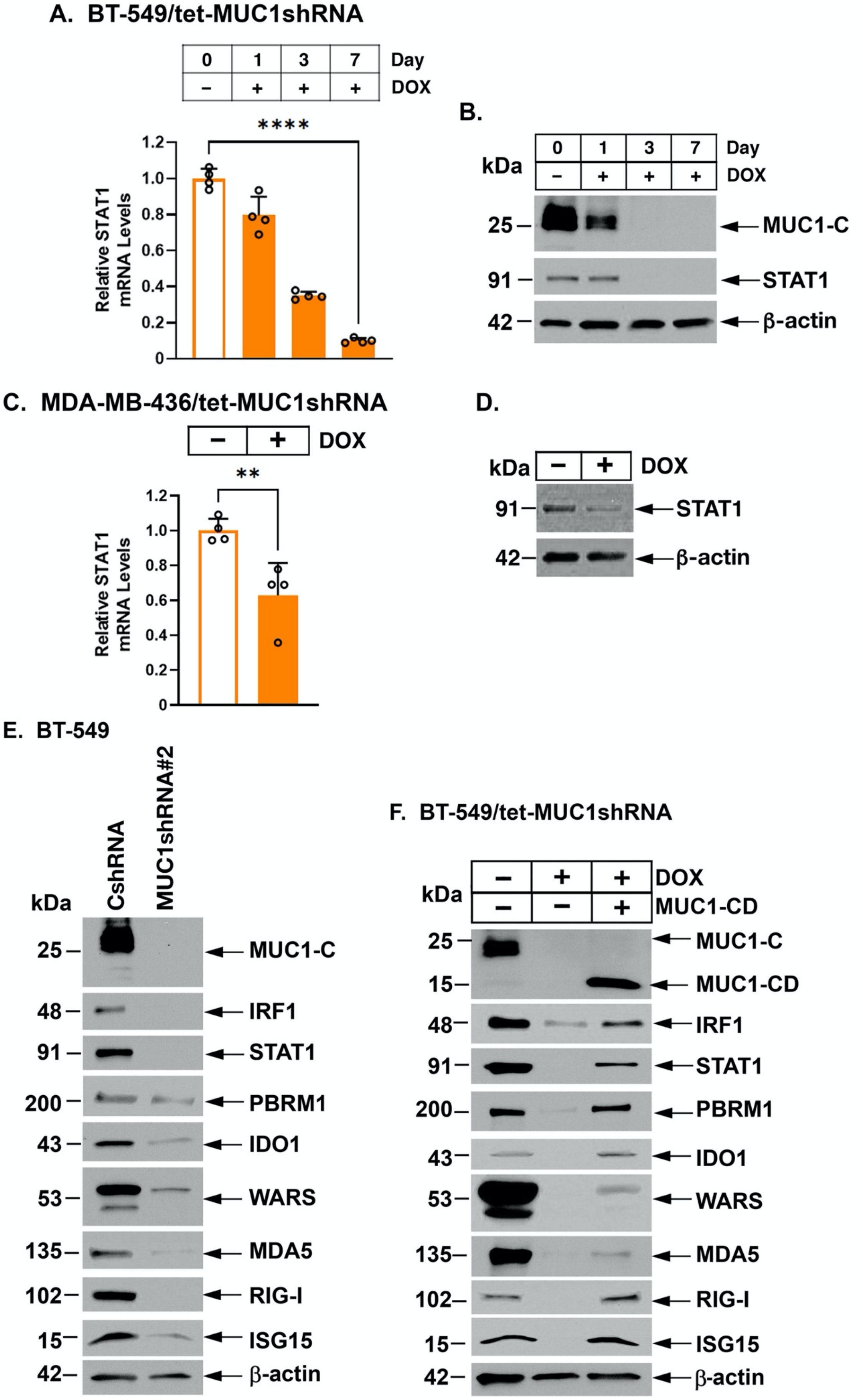

Supplemental Figure S2. Effects of silencing MUC1-C on TNBC cells. A and B. BT-549/tet-MUC1shRNA cells treated with vehicle or DOX for the indicated days were analyzed for STAT1 mRNA levels using

qRT-PCR (**A**). The results (mean±SD and individual values) are expressed as relative mRNA levels compared to those obtained in the control vehicle-treated cells (assigned a value of 1). Lysates were immunoblotted using antibodies against the indicated proteins (**B**). **C and D.** MDA-MB-436/tet-MUC1shRNA cells treated with vehicle or DOX for 7 days were analyzed for STAT1 mRNA levels using qRT-PCR (**C**). The results (mean±SD and individual values) are expressed as relative mRNA levels compared to those obtained in the control vehicle-treated cells (assigned a value of 1). Lysates were immunoblotted using antibodies against the indicated proteins (**D**). **E.** Lysates from BT-549/CshRNA and BT-549/MUC1shRNA#2 cells were immunoblotted with antibodies against the indicated proteins. **F.** BT-549/tet-MUC1shRNA cells were transfected to express the tet-MUC1-C cytoplasmic domain (MUC1-CD) and treated with the vehicle or DOX for 7 days. Lysates were immunoblotted using antibodies against the indicated proteins.

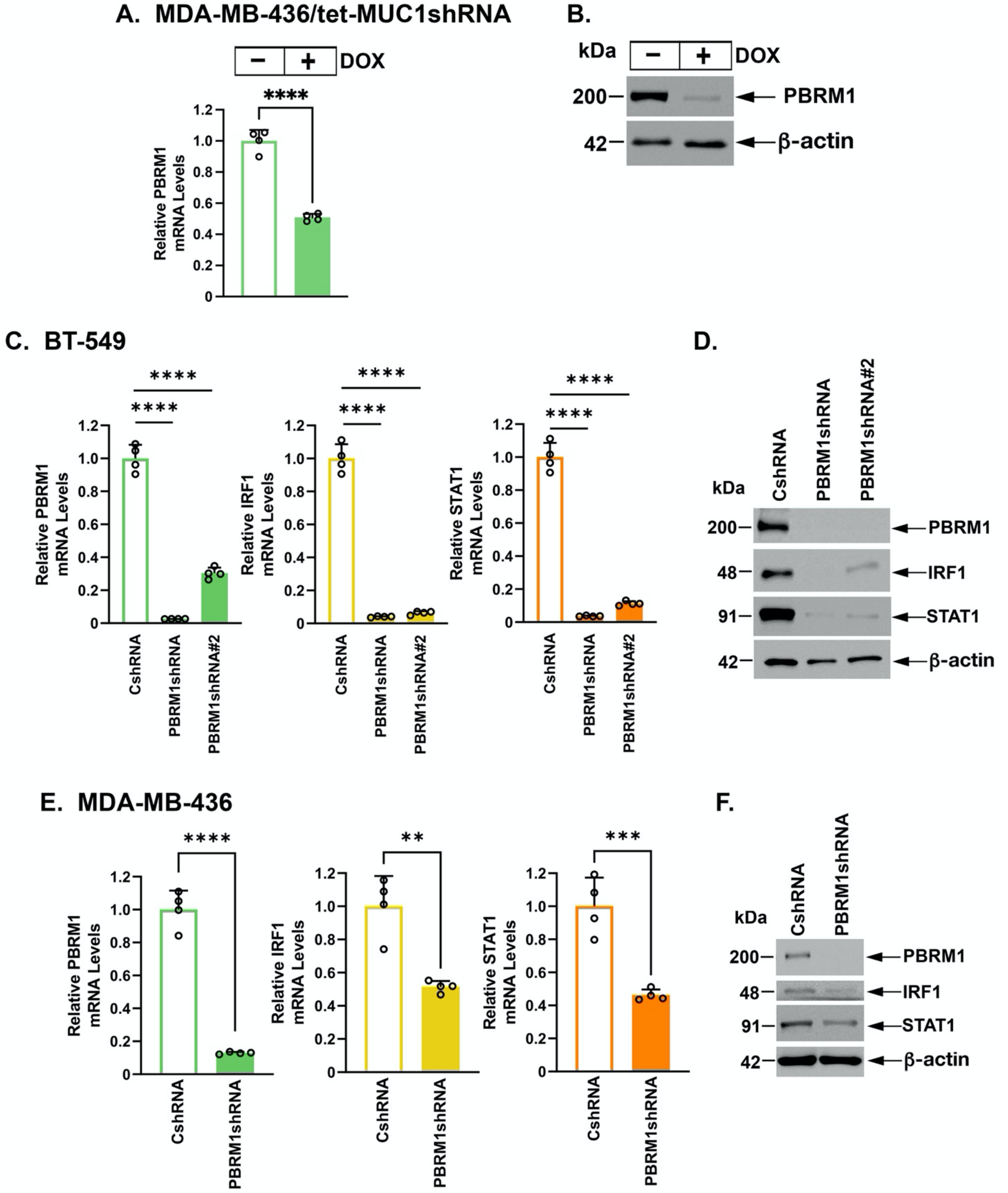

Supplemental Figure S3. MUC1-C is necessary for PBRM1 expression A and B. MDA-MB-436/tet-MUC1shRNA cells treated with vehicle or DOX for 7 days were analyzed for PBRM1 mRNA levels using qRT-PCR (**A**). The results (mean±SD and individual values) are expressed as relative mRNA levels compared to those obtained in the control vehicle-treated cells (assigned a value of 1). Lysates were immunoblotted using antibodies against the indicated proteins (**B**). **C and D.** BT-549/CshRNA, BT-549/PBRM1shRNA, and BT-549/PBRM1shRNA#2 cells were analyzed for PBRM1, IRF1 and STAT1 mRNA levels by qRT-PCR (**C**). The results (mean±SD and individual values) are expressed as relative mRNA levels compared to those obtained in the control vehicle-treated cells (assigned a value of 1). Lysates were

immunoblotted using antibodies against the indicated proteins (**D**). **E and F.** MDA-MB-436/CshRNA and MDA-MB-436/PBRM1shRNA cells were analyzed for PBRM1, IRF1, and STAT1 mRNA levels using qRT-PCR (**E**). The results (mean±SD and individual values) are expressed as relative mRNA levels compared to those obtained in the control vehicle-treated cells (assigned a value of 1). Lysates were immunoblotted using antibodies against the indicated proteins (**F**).

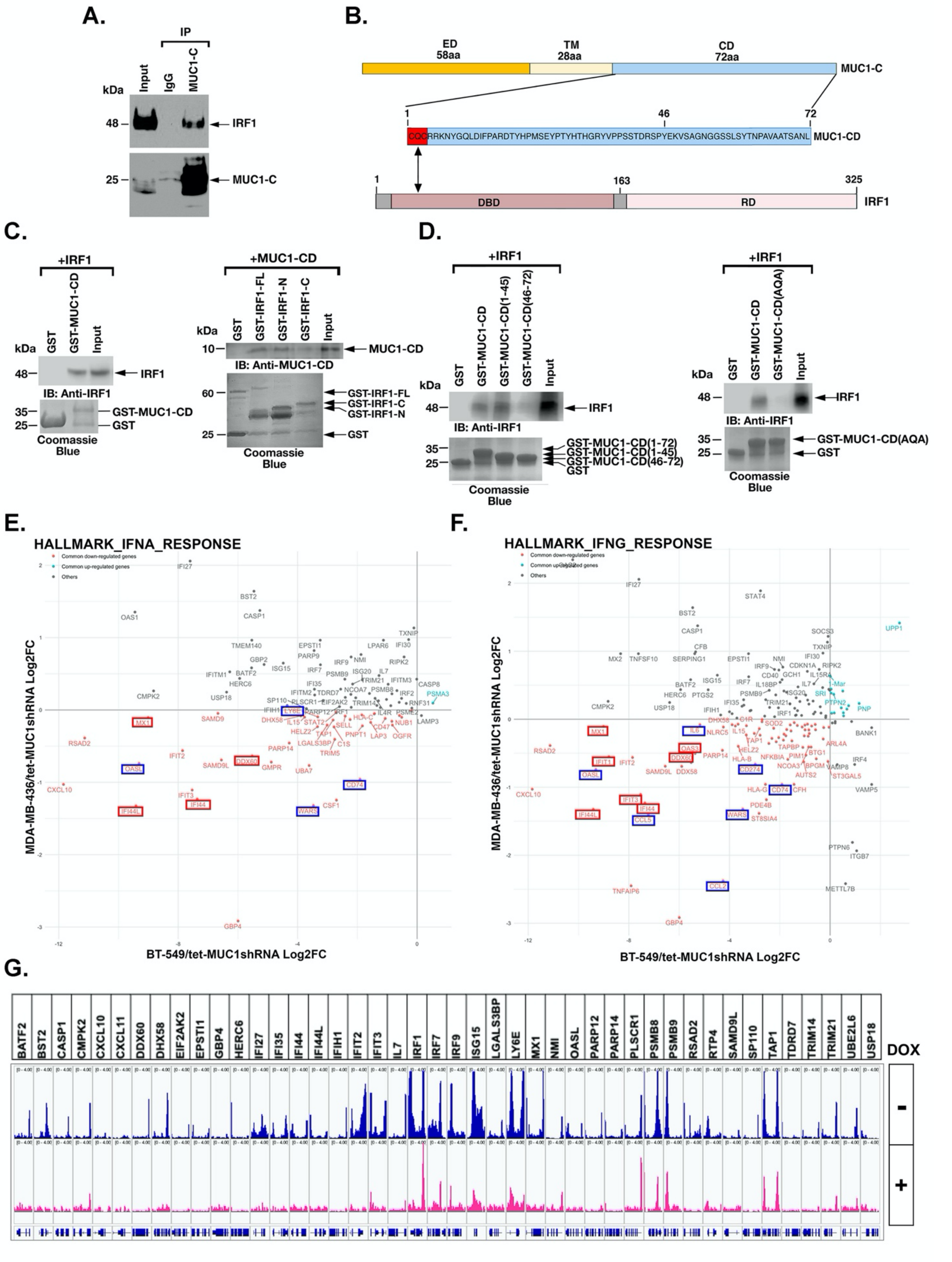

Supplemental Figure S4. MUC1-C forms a direct complex with IRF1 that activates type I and II ISGs. A. Nuclear lysates from BT-549 cells were precipitated with control IgG or anti-MUC1-C. Input proteins and precipitates were immunoblotted using antibodies against the indicated proteins. **B.** Schema of the MUC1-C protein with the amino acid sequence of the 72 aa cytoplasmic domain. Schema of the IRF1 325 aa protein highlighting regions of the DNA-binding (aa

1-163) and regulatory (aa 163-325) domains. **C.** GST and GST-MUC1-CD(FL; 1-72 aa) were incubated with purified IRF1(1-325)(left). The adsorbates and inputs were immunoblotted using anti-IRF1 antibody. GST, GST-IRF1(1-325), GST-IRF1-N(1-163) and GST-IRF1-C(163-325) were incubated with purified MUC1-CD(1-72)(right). The adsorbates and inputs were immunoblotted using an anti-MUC1-CD antibody. The input of GST proteins was assessed using Coomassie blue staining. **D.** GST, GST-MUC1-CD(FL; 1-72 aa), GST-MUC1-CD(1-45) and GST-MUC1-CD(45-72) were incubated with purified IRF1(1-325)(left). GST, GST-MUC1-CD, and GST-MUC1-CD(CQC to AQA mutation) were incubated with purified IRF1(1-325)(right). The adsorbates and inputs were immunoblotted using anti-IRF1 antibody. The input of GST proteins was assessed using Coomassie blue staining. **E and F.** Common MUC1-activated and -repressed genes in BT-549/tet-MUC1shRNA and MDA-MB-436/tet-MUC1shRNA cells treated with DOX for 7 days using HALLMARK IFNA (**E**) and IFNG (**F**) RESPONSE gene signatures. G. Genome browser snapshots of ATAC-seq data of 44 common MUC1-, IRF1-, and PBRM1-regulated genes in BT-549/tet-MUC1shRNA cells treated with vehicle or DOX for 7 days.

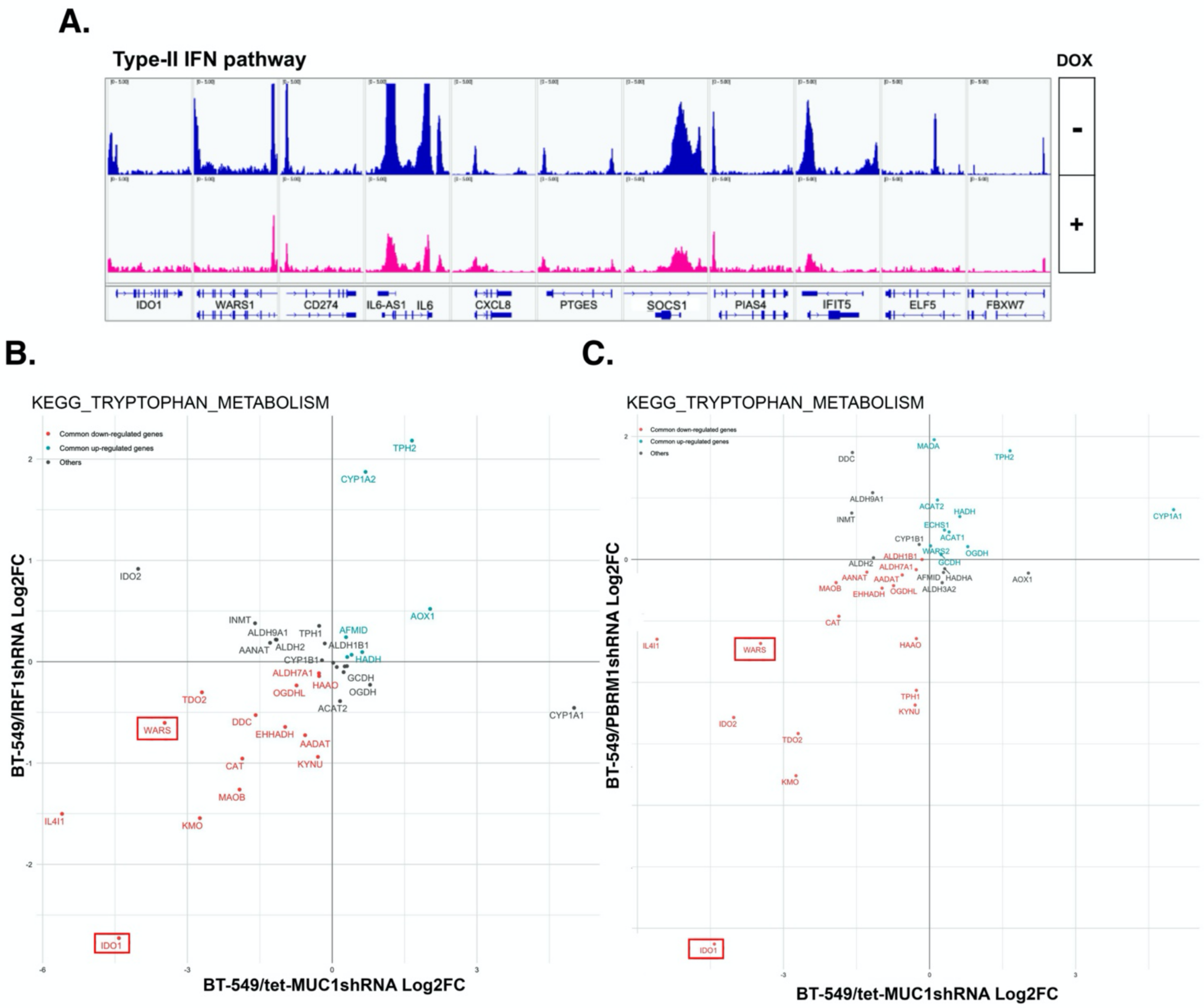

Supplemental Figure S5. Common genes regulated in cells silenced for MUC1, IRF1, and PBRM1. A. Genome browser snapshots of ATAC-seq data from the indicated type II IFN genes in BT-549/tet-MUC1shRNA cells treated with vehicle or DOX for 7 days. **B.** Common down- and upregulated genes in BT-549 cells with MUC1 and IRF1 silencing obtained from GSEA of the KEGG TRYPTOPHAN METABOLISM gene signature. **C.** Common down- and upregulated genes in BT-549 cells with MUC1 and PBRM1 silencing obtained from GSEA of the KEGG TRYPTOPHAN METABOLISM gene signature. Highlighted are IDO1 and WARS, which were downregulated by MUC1-C, IRF1, and PBRM1 silencing.

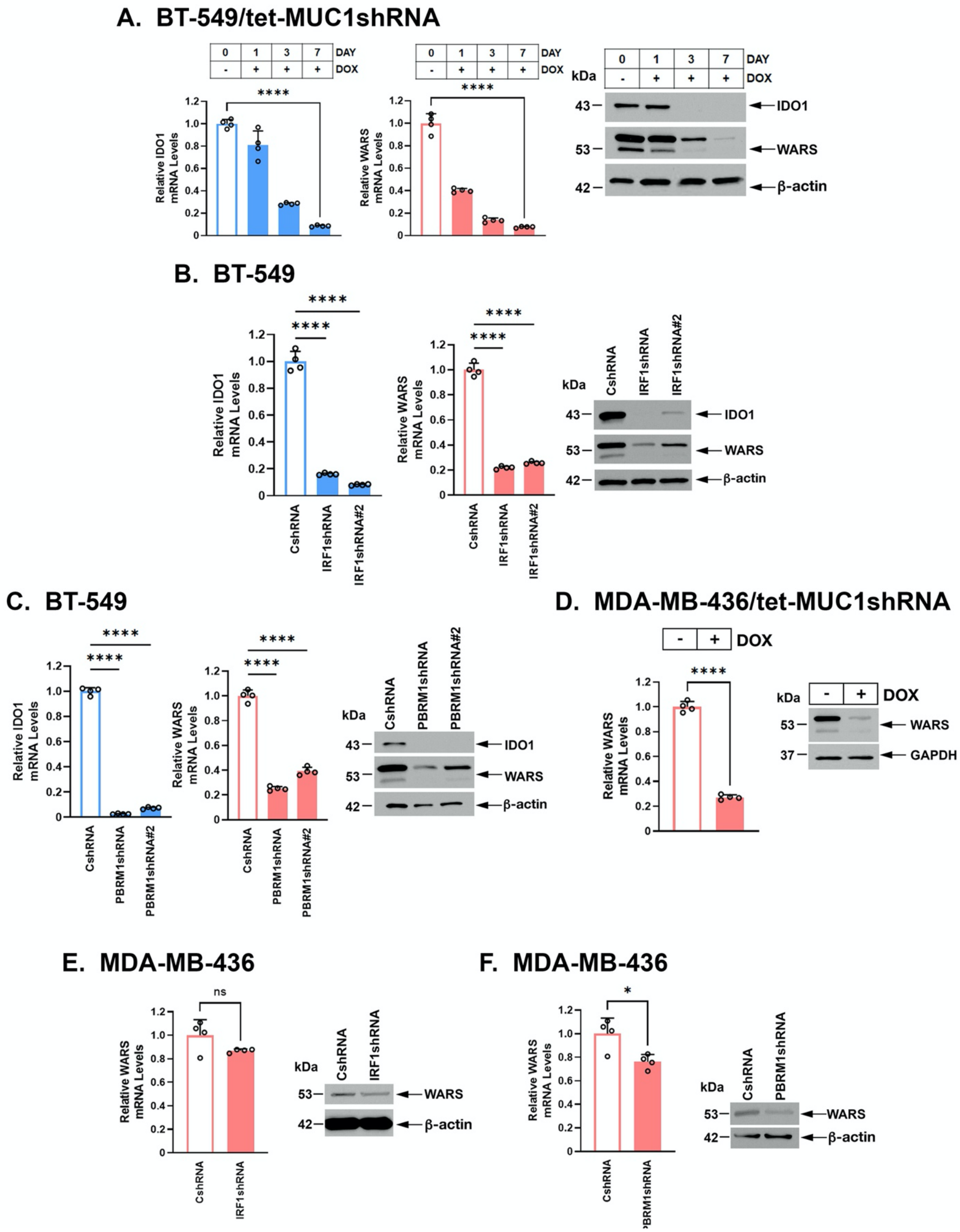

Supplemental Figure S6. Silencing MUC1-C, IRF1, and PBRM1 downregulated IDO1 and WARS in TNBC cells. A. BT-549/tet-MUC1shRNA cells treated with vehicle or DOX for the indicated days were analyzed for IDO1 and WARS mRNA levels using qRT-PCR (left). The results (mean±SD and individual values) are expressed as relative mRNA levels compared to those obtained in the control-treated cells (assigned a value of 1). Lysates were immunoblotted using antibodies against the indicated proteins (right). **B.** BT-549/CshRNA, BT-549/IRF1shRNA, and BT-549/IRF1shRNA#2 cells were analyzed for IDO1 and WARS mRNA levels using qRT-PCR (left). The results (mean±SD and individual values) are expressed as relative mRNA levels compared

with those obtained in CshRNA cells (assigned a value of 1). Lysates were immunoblotted using antibodies against the indicated proteins (right). **C.** BT-549/CshRNA, BT-549/PBRM1shRNA, and BT-549/PBRM1shRNA#2 cells were analyzed for IDO1 and WARS mRNA levels using qRT-PCR (left). The results (mean±SD and individual values) are expressed as relative mRNA levels compared with those obtained in CshRNA cells (assigned a value of 1). Lysates were immunoblotted using antibodies against the indicated proteins (right). **D.** MDA-MB-436/tet-MUC1shRNA cells treated with vehicle or DOX for the indicated days were analyzed for WARS mRNA levels using qRT-PCR (left). The results (mean±SD and individual values) are expressed as relative mRNA levels compared with those obtained in vehicle-treated cells (assigned a value of 1). Lysates were immunoblotted using antibodies against the indicated proteins (right). **E.** MDA-MB-436/CshRNA, MDA-MB-436/IRF1shRNA, and MDA-MB-436/IRF1shRNA#2 cells were analyzed for WARS mRNA levels using qRT-PCR (left). The results (mean±SD and individual values) are expressed as relative mRNA levels compared with those obtained in CshRNA cells (assigned a value of 1). Lysates were immunoblotted using antibodies against the indicated proteins (right). **F.** MDA-MB-436/CshRNA and MDA-MB-436/PBRM1shRNA cells were analyzed for WARS mRNA levels using qRT-PCR (left). The results (mean±SD and individual values) are expressed as relative mRNA levels compared with those obtained in CshRNA cells (assigned a value of 1). Lysates were immunoblotted using antibodies against the indicated proteins (right).

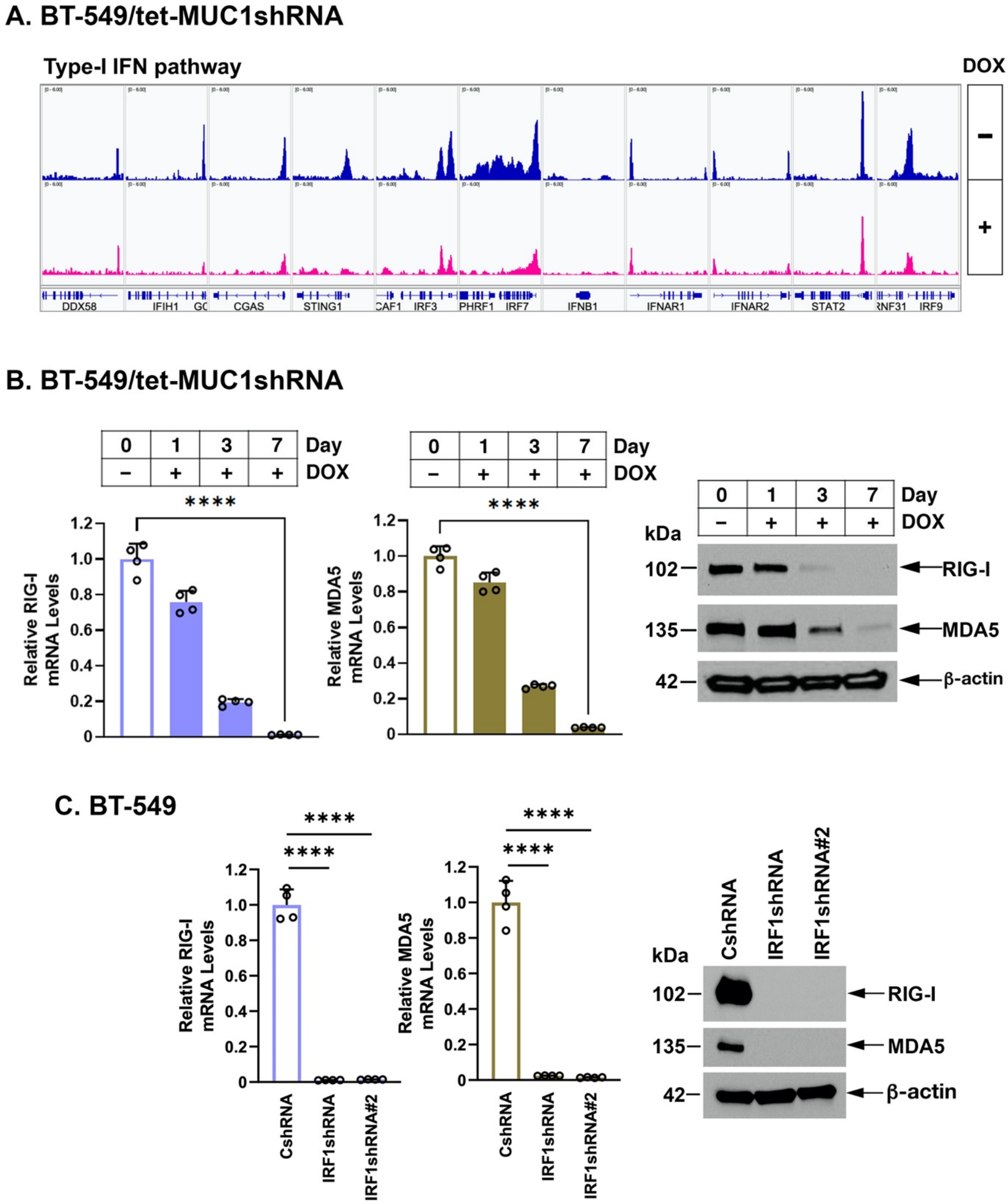

Supplemental Figure S7. Silencing of MUC1-C, IRF1, and PBRM1 downregulates RIG-I and MDA5 in TNBC cells. A. Genome browser snapshots of ATAC-seq data from the indicated type I IFN genes in BT-549/tet-MUC1shRNA cells treated with vehicle or DOX for 7 days. **B.** BT-549/tet-MUC1shRNA cells treated with vehicle or DOX for the indicated days were analyzed for RIG-I and MDA5 mRNA levels using qRT-PCR (left). The results (mean±SD and individual values) are expressed as relative mRNA levels compared with those obtained in vehicle-treated cells (assigned a value of 1). Lysates were immunoblotted using antibodies against the indicated proteins (right). **C.** BT-549/CshRNA, BT-549/IRF1shRNA, and BT-549/IRF1shRNA#2 cells were analyzed for RIG-I and MDA5 mRNA levels using qRT-PCR (left). The results (mean±SD and individual values) are expressed as relative mRNA levels compared with those obtained in CshRNA cells (assigned a value of 1). Lysates were immunoblotted using antibodies

against the indicated proteins (right). **D.** BT-549/CshRNA, BT-549/PBRM1shRNA, and BT-549/PBRM1shRNA#2 cells were analyzed for RIG-I and MDA5 mRNA levels using qRT-PCR (left). The results (mean±SD and individual values) are expressed as relative mRNA levels compared with those obtained in CshRNA cells (assigned a value of 1). Lysates were immunoblotted using antibodies against the indicated proteins (right). **E.** MDA-MB-436/tet-MUC1shRNA cells treated with vehicle or DOX for the indicated number of days were analyzed for RIG-I and MDA5 mRNA levels by qRT-PCR (left). The results (mean±SD and individual values) are expressed as relative mRNA levels compared with those obtained in vehicle-treated cells (assigned a value of 1). Lysates were immunoblotted using antibodies against the indicated proteins (right). **F.** MDA-MB-436/CshRNA, MDA-MB-436/IRF1shRNA and MDA-MB-436/IRF1shRNA#2 cells were analyzed for RIG-I and MDA5 mRNA levels by qRT-PCR (left). The results (mean±SD and individual values) are expressed as relative mRNA levels compared with those obtained in CshRNA cells (assigned a value of 1). Lysates were immunoblotted using antibodies against the indicated proteins (right). **G.** MDA-MB-436/CshRNA, MDA-MB-436/PBRM1shRNA and MDA-MB-436/PBRM1shRNA#2 cells were analyzed for RIG-I and MDA5 mRNA levels by qRT-PCR (left). The results (mean±SD and individual values) are expressed as relative mRNA levels compared with those obtained in CshRNA cells (assigned a value of 1). Lysates were immunoblotted using antibodies against the indicated proteins (right).

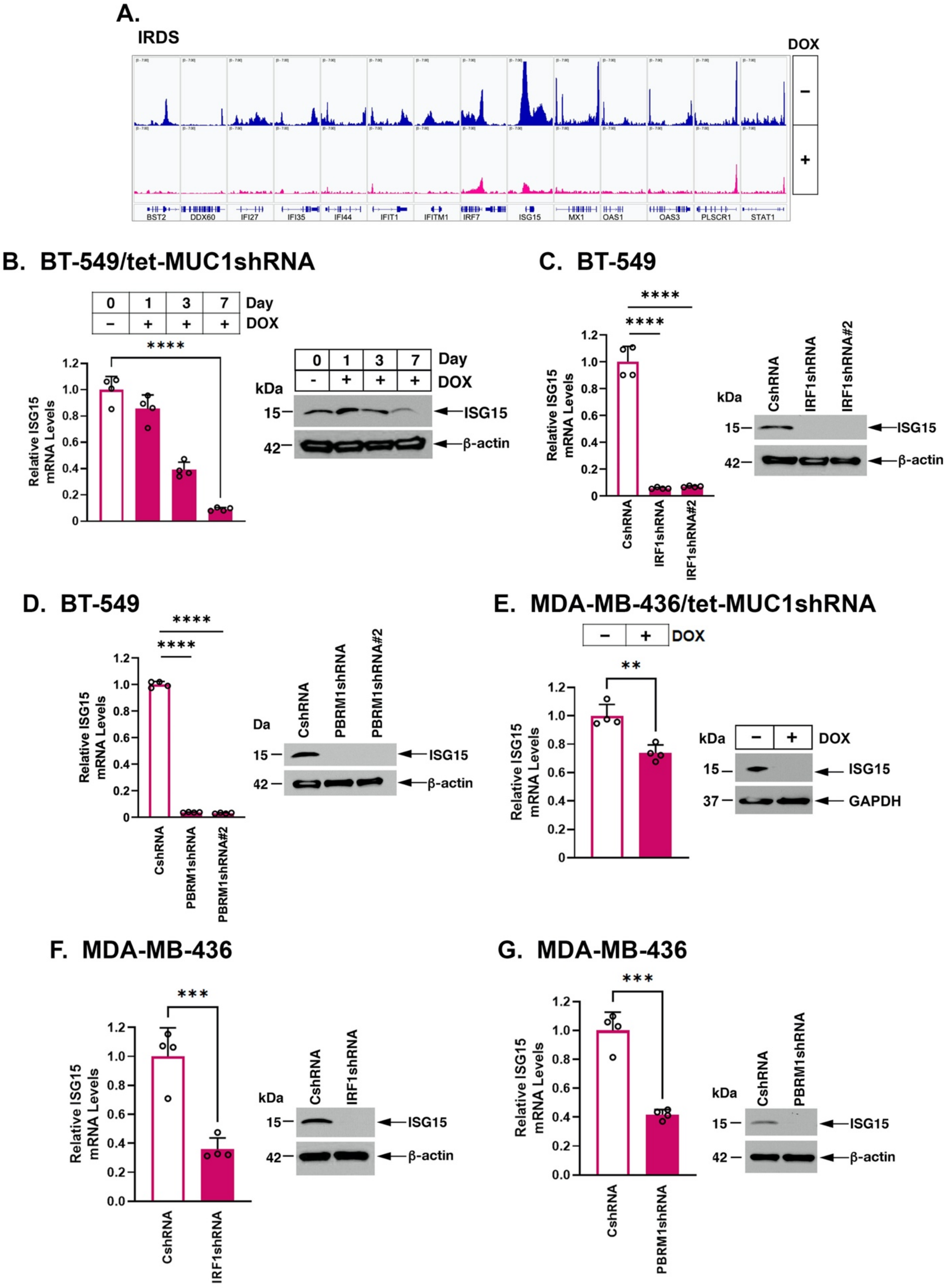

Supplemental Figure S8. Silencing MUC1-C, IRF1, and PBRM1 downregulates ISG15 expression in TNBC cells. A. Genome browser snapshots of ATAC-seq data from the indicated IRDS genes in BT-549/tet-MUC1shRNA cells treated with vehicle or DOX for seven days. **B.** BT-549/tet-MUC1shRNA cells treated with vehicle or DOX for the indicated days were analyzed for ISG15 mRNA levels using qRT-PCR (left). The results (mean±SD and individual values) are expressed as

relative mRNA levels compared with those obtained in vehicle-treated cells (assigned a value of 1). Lysates were immunoblotted using antibodies against the indicated proteins (right). **C.** BT-549/CshRNA, BT-549/IRF1shRNA, and BT-549/IRF1shRNA#2 cells were analyzed for ISG15 mRNA levels using qRT-PCR (left). The results (mean±SD and individual values) are expressed as relative mRNA levels compared with those obtained in CshRNA cells (assigned a value of 1). Lysates were immunoblotted using antibodies against the indicated proteins (right). **D.** BT-549/CshRNA, BT-549/PBRM1shRNA, and BT-549/PBRM1shRNA#2 cells were analyzed for ISG15 mRNA levels using qRT-PCR (left). The results (mean±SD and individual values) are expressed as relative mRNA levels compared with those obtained in CshRNA cells (assigned a value of 1). Lysates were immunoblotted using antibodies against the indicated proteins (right). **E.** MDA-MB-436/tet-MUC1shRNA cells treated with vehicle or DOX for the indicated number of days were analyzed for ISG15 mRNA levels by qRT-PCR (left). The results (mean±SD and individual values) are expressed as relative mRNA levels compared with those obtained in vehicle-treated cells (assigned a value of 1). Lysates were immunoblotted using antibodies against the indicated proteins (right). **F.** MDA-MB-436/CshRNA, MDA-MB-436/IRF1shRNA, and MDA-MB-436/IRF1shRNA#2 cells were analyzed for ISG15 mRNA levels using qRT-PCR (left). The results (mean±SD and individual values) are expressed as relative mRNA levels compared with those obtained in CshRNA cells (assigned a value of 1). Lysates were immunoblotted using antibodies against the indicated proteins (right). **G.** MDA-MB-436/CshRNA, MDA-MB-436/PBRM1shRNA, and MDA-MB-436/PBRM1shRNA#2 cells were analyzed for ISG15 mRNA levels using qRT-PCR (left). The results (mean±SD and individual values) are expressed as relative mRNA levels compared with those obtained in CshRNA cells (assigned a value of 1). Lysates were immunoblotted using antibodies against the indicated proteins (right).

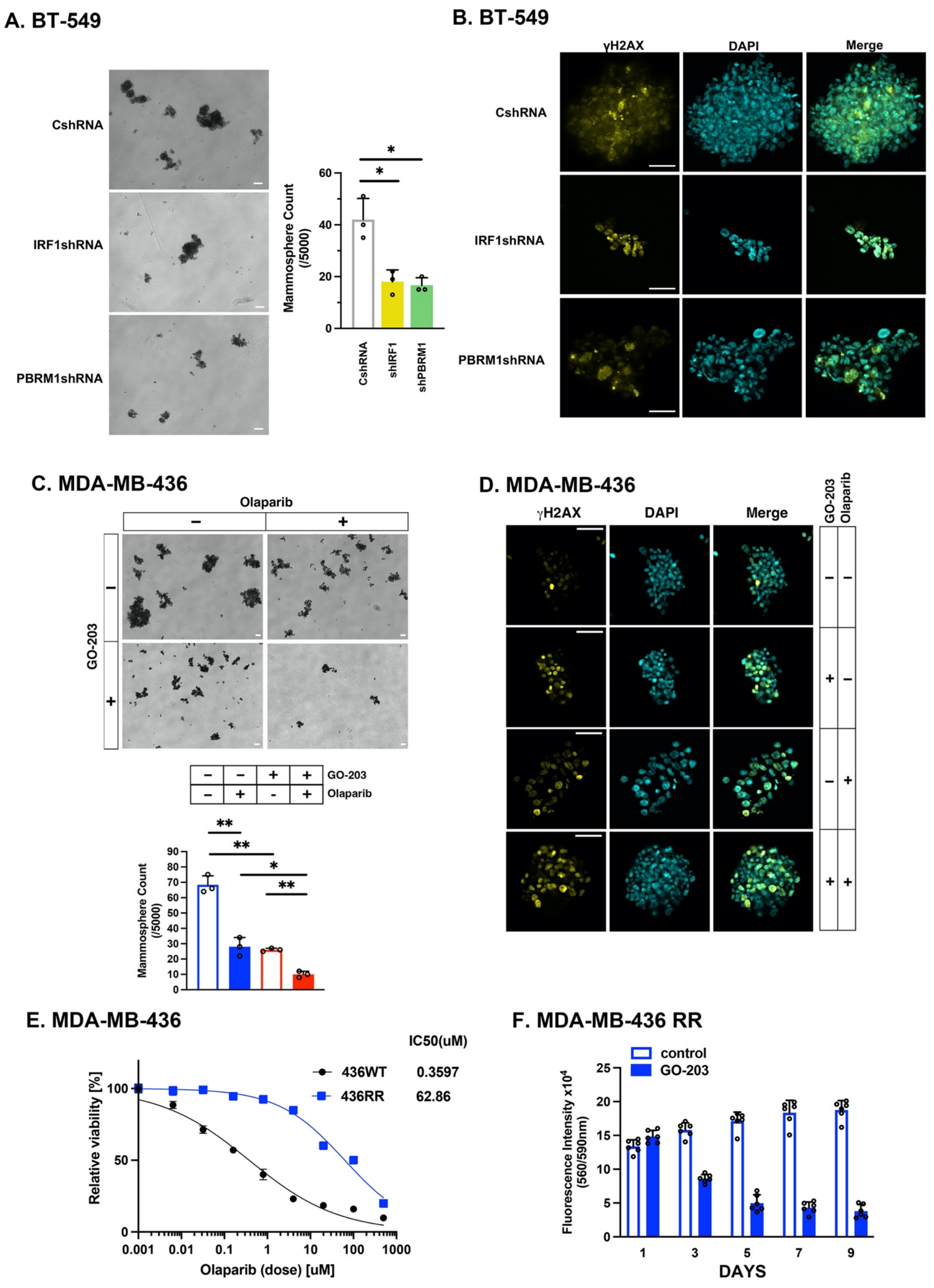

Supplemental Figure S9. Effects of silencing IRF1 and PBRM1 on BT-549 mammosphere formation and GO-203 treatment on the viability of olaparib-resistant MDA-MB-436RR cells. A and B. BT-549/CshRNA, BT-549/IRF1shRNA, and BT-549/PBRM1shRNA cells were assayed for mammosphere formation on day 7 (**A**, left). Scale bar: 100 μm. The

results (mean ± SD of three biological replicates) are expressed as the number of mammospheres (**A**, right panel). The indicated mammospheres were assayed by ICC analysis to visualize γH2AX foci (**B**). Scale bar:50 μm. **C.** MDA-MB-436 cells treated with vehicle or 1 μM GO-203 in the absence and presence of 5 μM olaparib were assayed for mammosphere formation at 8 days (left). Scale bar: 100 μm. The results (mean+SD of 3 biological replicates) are expressed as the number of mammospheres (right). **D.** MDA-MB-436 mammospheres treated with vehicle or 2.5 μM GO-203 in the absence and presence of 1 μM for 2 days were assayed by ICC analysis for visualization of γH2AX foci. Scale bar:50 μm. **E.** MDA-MB-436 and MDA-MB-436RR cells were treated with olaparib at the indicated concentrations for five days. Cell viability was determined using the Alamar Blue assay. **F.** MDA-MB-436RR cells were treated with 5 *μ*M GO-203 for the indicated times. Fluorescence intensity (560/590 nm) was expressed as the mean ± SD of six measurements.

Table S1. Primers used for qRT-PCR.

Primer	FWD	REV
**MUC1-C**	AGACGTCAGCGTGAGTGATG	GCCAAGGCAATGAGATAGAC
**PBRM1**	AAGAAGAAAGAGCTTGCCAG	TCTCGAGCTTCAAGAACAAC
**ARID2**	GCAGCCAATTTCCACTCCTGTTG	GATTGGTGACAGGAGTCCTCTG
**STAT1**	GGAACTTGATGGCCCTAAAGGA	ACAGAGCCCACTATCCGAGACA
**IRF1**	CATGGCTGGGACATCAACAA	TTGTATCGGCCTGTGTGAATG
**IDO1**	TCTCATTTCGTGATGGAGACT	GTGTCCCGTTCTTGCATTTGC
**WARS**	TGACGGATGACGAGAAGTATCT	GCCGAAAATGCCTTTCACTTG
**RIG-I**	CTGGACCCTACCTACATCCTG	GGCATCCAAAAAGCCACGG
**MDA5**	CCATGGAGAAGGCTGGGG	CAAAGTTGTCATGGATGACC
**ISG15**	CGCAGATCACCCAGAAGATCG	TTCGTCGCATTTGTCCACCA
**β-actin**	GATGAGATTGGCATGGCTTT	CACCTTCACCGTTCCAGTTT

Supplemental Table S2. Primers used for ChIP-qPCR and DNase I chromatin accessibility assays.

Primer	FWD	REV
**pIRF1_dELS_DN**	TGCTGGGTAGTGTTTATGCC	TGGAAGGTGTGGATATGTGC
**pIRF1_PLS**	TTCGCCGCTAGCTCTAC	GCCGCGCGGGCGCCCATT
**pIRF1_dELS_UP**	GAAAAGCCCCACCTGAATG	GTCCATCCTTCACACCCC
**pPBRM1_PLS**	GAACCGTCAAGAAACCACAAC	GTTTCCCTCAGTCCCCAATAC
**pSTAT1**	ATGCTTCCGAGCTGTCAAGT	TGTTGCTAAACCCAGGGAAC
**pIDO1**	TGCACAGAGATGCTTTTGTGG	GCCAGTGACCACAGTTTATCAC
**pWARS**	GAACAGATGCGGGGAATTTA	CGAGCCATTAGCTGGTCATT
**pRIG1**	GGAGGGAAACGAAACTAGCC	TTAAAGCCGGGTAGGAGGAG
**pMDA5**	CTTTGTAAACGTAATCTGCCTGG	GCTTTCCTTTTCTGTTTCCCG
**pISG15**	CGGTTTTGTTTCTTCCGCTCA	AGCACCGGCCCTATTATAAGC

Supplemental Table S3. Common downregulated 196 DEGs in BT-549 cells with silencing of MUC1, IRF1, and PBRM1.

CX3CL1	PLEKHA4	GALNT12	CMPK2	PPFIA4	IGFBP6	TCN2	HLA-E
ETV7	GIMAP2	IFIT3	OASL	GRIP2	TRANK1	SP140L	HLA-F
TNFRSF1B	TRIM14	IFIT2	LMO2	PLEKHS1	BATF2	IRF7	PSMB10
EIF2AK2	CA9	TNFSF10	SP110	SERPING1	TAP1	IFIT1	SAMD9
PARP12	DDX58	GLIPR2	PHF11	CCDC3	MLKL	TRIM69	KRT81
LZTS1	ACTA2	EGR2	GPNMB	IFIT5	HR	IFITM1	GBP7
SP100	MAP3K8	NMI	IL6	GBP5	PCSK9	BTN3A2	IRF9
IFI35	LGALS3BP	KCNJ2	ADAMTS7	UBE2L6	CXCL10	PLCD1	SMTNL1
TP63	DHX58	IRF1	RTP4	KCNJ15	CXCL11	ISG15	PLXNA4
CA12	MAP2K6	IFI6	FHDC1	MX1	LRRN2	HES4	APOL6
ACTN2	SLC15A3	APOL3	LRRC32	ZNF618	MYD88	PLSCR1	GBP1P1
MOXD1	OAS3	APOBEC3F	DDX60	TNFRSF14	PARP14	H1F0	NRIR
IGSF9	OAS2	ISLR	CASP1	LY6E	FZD4	PAX5	RPS6KA2-IT1
OAS1	BTN3A3	APOE	IFI44L	USP41	SAMD9L	TDRD7	LINC02574
TBX15	MDGA1	BST2	IFI44	CXCL16	CALHM5	ARL9	LINC01914
IL12RB1	TRIM38	SHFL	CH25H	GBP4	PARP10	ACSL5	HLA-B
TSPAN15	ST8SIA4	IDO1	PARP9	IGFBP7	PCGF5	UAP1L1	APOBEC3G
APOL1	PLSCR4	TRIM21	HERC6	DTX3L	MUC16	SLC28A3	PSMB9
MYL9	IFIH1	TRIM22	HERC5	ERAP2	DDX60L	C5orf56	LY6E-DT
TLDC2	GCA	XAF1	PRKG2	TLR3	TNFSF15	PLCG2	CCL5
SAMHD1	STAT1	ACY3	NLRC5	HNF4G	UBA7	TMEM229B	BISPR
QPRT	KMO	KANK4	PMAIP1	KCNV1	BGN	IFIT1B	
IL7	GBP3	EPSTI1	PGGHG	AQP3	TMEM173	PSMB8	
FCGRT	GBP1	PLAAT4	IL22RA1	IFI27	RBM43	COL15A1	
IL4I1	CDK18	RSAD2	LHX9	B2M	USP18	CARD16	

Supplemental Table S4. Overlapping IFN-pathway genes downregulated in cells with MUC1-C, PBRM1, and IRF1 silencing.

B2M
BATF2
BST2
CASP1
CMPK2
CXCL10
CXCL11
DDX60
DHX58
EIF2AK2
EPSTI1
GBP4
HERC6
IFI27
IFI35
IFI44
IFI44L
IFIH1
IFIT2
IFIT3
IL7
IRF1
IRF7
IRF9
ISG15
LGALS3BP
LY6E
MX1
NMI
OASL
PARP12
PARP14
PLSCR1
PSMB8
PSMB9
RSAD2
RTP4
SAMD9L
SP110
TAP1
TDRD7
TRIM14
TRIM21
UBE2L6
USP18
